# Beyond Molecular Structures: Investigating Demographic Factors in Drug-Induced Cardiotoxicity Prediction Models

**DOI:** 10.1021/acs.jcim.6c00418

**Published:** 2026-06-02

**Authors:** Mateusz Iwan, Alessandra Roncaglioni, Francesca Grisoni

**Affiliations:** † Department of Biomedical Engineering, Eindhoven University of Technology, Institute for Complex Molecular Systems (ICMS), P.O. Box 513, Eindhoven 5600 MB, The Netherlands; ‡ Department of Environmental Health Sciences, Istituto di Ricerche Farmacologiche Mario Negri IRCCS, Via Mario Negri 2, Milan 20156, Italy

## Abstract

Predicting drug-induced cardiotoxicity remains one of the most important challenges in drug safety, contributing to a substantial share of clinical trial failures and postmarket withdrawals. While clinical evidence shows differences in adverse responses across sex, age, and body mass, incorporating demographic factors into in silico prediction models remains challenging. We developed CARBIDE (CARdiotoxicity Based on Integrated Demographic Evidence), a collection of 27 dataset variants derived from the FAERS pharmacovigilance database, to systematically evaluate whether meaningful structure-demographic interactions could be learned from spontaneous reporting data. Through systematic evaluation of different FAERS filtering criteria, cardiotoxicity definitions, and statistical methods, together with comprehensive ablation studies, we found that machine learning models failed to extract useful structure-demographic relationships. The models either learned population-level statistics or relied solely on structural information, with demographic features derived using our approach providing little additional predictive value. While these findings reveal fundamental limitations in using pharmacovigilance data for demographic-aware toxicity prediction, CARBIDE’s systematic evaluation provides important insights for the field, helping guide future efforts toward more effective approaches in personalized cardiotoxicity prediction.

## Graphical abstract

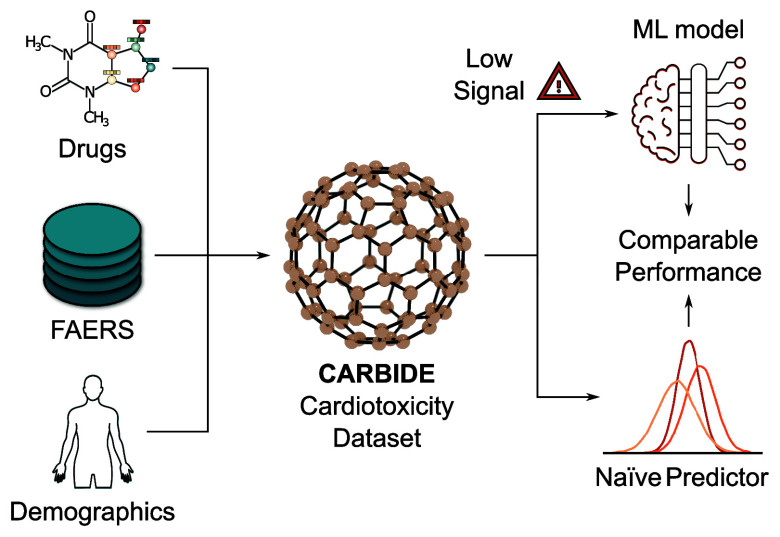

## 1 Introduction

Drug-induced cardiotoxicity (DICT) represents a significant challenge in drug development, accounting for approximately 14% of all postmarket withdrawals.^1^ Notable examples include rofecoxib, a selective cyclooxygenase-2 inhibitor removed due to a nearly 2-fold increase in serious cardiovascular events,^2^ and sibutramine, a weight loss drug removed from European markets due to an elevated risk of myocardial infarction.^3^

DICT encompasses various cardiac adverse effects, including arrhythmia, changes in contractility, hypertension and hypotension, and direct damage to cardiomyocytes.^4,5^ The associated risk is influenced by several demographic factors that are not yet fully characterized. Current evidence suggests that women are at increased risk of developing arrhythmia due to increased cardiac electrical changes at equivalent doses.^6^ Elderly patients show altered pharmacokinetics, including elevated plasma concentrations and decreased renal drug elimination, resulting in higher rates of cardiotoxic events.^7^ Body mass further complicates the cardiotoxicity risk due to obesity-associated comorbidities and altered drug distribution.^8^

Machine learning (ML) has been widely applied to predict chemical toxicity,^9,10^ including DICT in its various forms: cardiac ion channel interactions, clinical arrhythmias, and direct effects on cardiomyocytes.^11–16^ Among the available resources for DICT prediction, the DICTrank dataset stands out by providing structured general cardiotoxicity risk annotations derived from FDA labeling documents.^17^ It has since been used to train several ML models of cardiotoxicity.^18,19^

Despite the abundance of DICT prediction models, existing approaches do not account for the demographic variability in cardiotoxic responses, implicitly assuming that all patients react similarly to medication, regardless of their sex, age, or weight. To address this gap, we (a) develop a novel dataset, CARBIDE (CARdiotoxicity Based on Integrated Demographic Evidence), derived from the FDA Adverse Event Reporting System (FAERS),^20^ and (b) conduct a systematic evaluation of ML models trained on structural and demographic features to predict cardiotoxicity risk. Using this approach, we provide important insights for the development of more effective personalized frameworks for DICT prediction and drug safety evaluation.

## 2 Results and Discussion

### 2.1 Data Preparation and Characterization

We processed drug descriptions from the FAERS database (2012–2024),^20^ mapping them to 2970 unique chemical structures encoded as the Simplified Molecular Input Line Entry System (SMILES),^21^ and categorized demographic information (sex, age, and weight) into standardized groups to construct CARBIDE.

We built multiple CARBIDE variants by applying different FAERS filtering criteria (“Mono”, “Primary”, and “Secondary”), cardiotoxicity definitions (three sets of Medical Dictionary for Regulatory Activities (MedDRA) Preferred Terms (PTs) of increasing scope: “Cred”, “Card”, and “Cvas”^22^), and Disproportionality Analysis (DPA) metrics (Proportional Reporting Ratio (PRR), Reporting Odds Ratio (ROR), and Information Component (IC)).^23–26^ Entries were assigned to one of four cardiotoxicity risk classes based on relative positions of Confidence Intervals (CIs) to the metrics’ thresholds, with labels subsequently binarized into Toxic and Non-Toxic categories for initial modeling.

CARBIDE design choices had varying effects on the composition of each variant (Supporting Information, Table S1), as follows:

- The filtering criteria had the most substantial impact on the characteristics of the dataset. As the rules loosened (i.e., Secondary > Primary > Mono) the datasets became larger (15,000–20,000 entries for Mono vs 37,000–46,000 entries for Secondary), contained more unique compounds (∼1000 for Mono vs ∼1900 for Secondary), and had a slightly higher proportion of cardiotoxic labels.
- Expanding the definition of cardiotoxicity from the most focused (Cred, 341 terms) to broader cardiovascular conditions (Cvas, 848 terms) moderately increased dataset size and compound diversity while maintaining stable class distributions.
- The choice of the DPA metric had a minimal effect on dataset size, but substantially influenced the confidence scores of the classes. IC-based datasets showed higher confidence in toxic classifications (29–45% vs 23–28% for Non-Toxic), while ROR-based datasets exhibited the opposite pattern (19–29% Toxic vs 41–53% Non-Toxic). PRR-based datasets demonstrated more balanced confidence scores between classes.
- The average demographic coverage, measured as the number of entries per SMILES (EpS), was comparable between the Primary and Secondary variants (21–26) but was substantially lower in the Mono variants (16–18), likely due to a smaller number of included entries.

### 2.2 Cardiotoxicity Prediction

For model development, we used three classical ML models: Logistic Regression (LR), Random Forest (RF), and XGBoost (XGB)^27–29^ with six types of molecular descriptors.^30–35^ The models were evaluated in a nested cross-validation scheme, using Butina clustering^36^ to split the data into structurally distinct folds. After preliminary evaluations, Harmonic mean of Recall and Specificity (HarmRS) was used to guide the optimization process, as it (a) yielded performance comparable to that of other metrics like ROC AUC and (b) more effectively prevented models from collapsing to single-class predictions. We conducted a comprehensive evaluation of the 27 CARBIDE variants to investigate the effectiveness of predictive modeling. Figure 1 shows the performance of trained models together with 95% CIs, evaluated using metrics of discriminative ability (ROC AUC and PRC AUC), classification balance (recall, specificity, and HarmRS), and calibration (Brier score and ECE), as defined in Supporting Information, Section 3.4.

**Figure 1:**
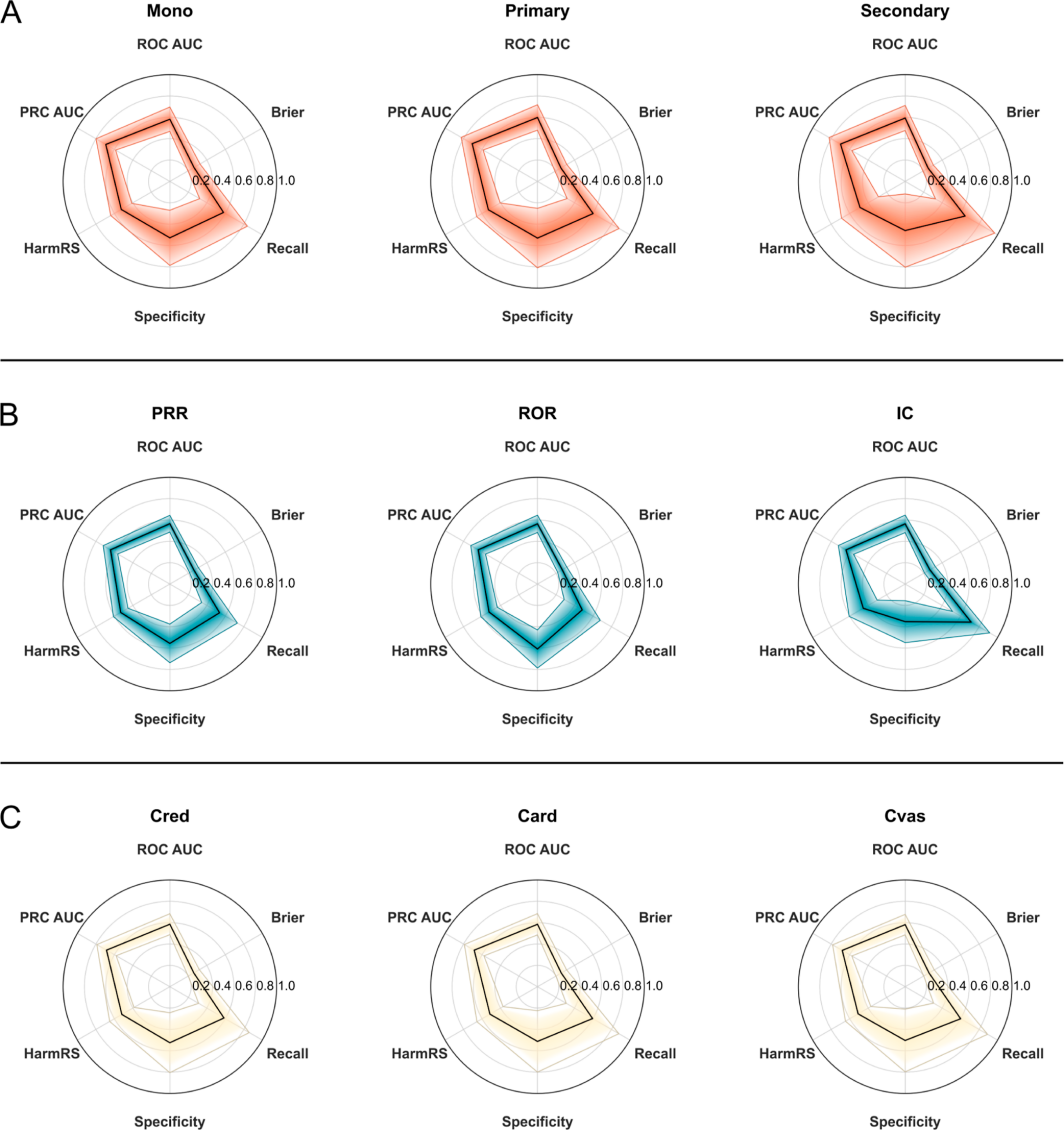
Radar plots summarizing predictive performance across CARBIDE variants, showing specificity, recall, Brier score, Receiver Operating Characteristic—Area Under Curve (ROC AUC), Precision Recall Curve—Area Under Curve (PRC AUC), and Harmonic mean of Recall and Specificity (HarmRS). Shaded regions represent 95% CIs. Results are aggregated by (A) FAERS filtering criteria, (B) DPA metric, (C) cardiotoxicity definition.

To quantify the differences between dataset variants, we performed pairwise statistical comparisons using Wilcoxon signed-rank tests^37^ with Holm-corrected *p*-values^38^ and matched-pairs rank–biserial correlation effect sizes (*r*_b_).^39^ Statistical comparisons across all dataset variants are provided in Supporting Information, Table S2. Exact performance metrics for filtering criteria, cardiotoxicity definitions, and DPA metrics are given in Supporting Information, Tables S3–S5.

#### 2.2.1 Filtering Criteria

Although the Secondary variants contain substantially more compounds, they did not yield improved predictive performance. Primary variants achieved small but statistically significant improvements over Secondary variants in both ROC AUC and PRC AUC. These likely indicate that including “Secondary Suspect” drugs in the DPA analysis degrades signal strength, which results in less balanced classification and worse model performance. Interestingly, restricting analysis to monotherapy cases (Mono variant) resulted in significantly worse performance, with medium effect sizes for both ROC AUC and PRC AUC compared to Primary variants. This could suggest that healthcare professionals’ assignment of “Primary Suspect” status effectively identifies the most relevant drug–event associations even in multidrug reports, providing more value than complete elimination of potential drug interactions.

#### 2.2.2 Cardiotoxicity Definition

Models trained on the Cred-based datasets achieved better performance than those trained on the broader Cvas-based datasets across both ROC AUC and PRC AUC, although with small effect sizes. Models trained on Card-based datasets showed similar improvements over Cvas, while differences between Cred and Card were negligible. These results may suggest that core cardiotoxicity signals are captured even by the most restrictive definition (Cred), with broader definitions potentially degrading the signal quality.

#### 2.2.3 DPA Metric

The choice of DPA metric had a minimal impact on model performance, with negligible effect sizes across all comparisons between PRR, ROR, and IC methods. However, the metrics showed substantial differences in the balance between recall and specificity. IC-based models exhibited strong bias toward predicting toxicity (high recall and poor specificity), ROR-based models showed the opposite pattern, while PRR-based models provided the most balanced performance. These tendencies likely stem from the distribution of confidence scores that were used as sample weights during training (Supporting Information, Table S1). From a practical perspective, the choice of the DPA metric represents a trade-off between different types of errors. IC-based models minimize false negatives at the expense of numerous false positives, ROR-based models do the opposite, and the PRR-based models provide a more balanced middle ground.

### 2.3 Agreement with DICTrank

To evaluate the label quality independent of demographic stratification, we compared the CARBIDE variants against the DICTrank dataset^17^ as a ground-truth reference. DICTrank was prepared using official FDA labeling documents for drugs and incorporates warnings about possible adverse drug reactions and their severity. The dataset contains predominantly cardiotoxic compounds (74%). The class-by-class comparison is presented in Figure 2. Although Secondary variants generally showed higher overall agreement rates (50–76%) compared to Primary (52–74%) and Mono (53–66%) variants, this occurs primarily because the majority of compounds were assigned to the Toxic class, which corresponds to general label trends in the DICTrank dataset. This comes at the cost of fewer true negatives; we suppose that the inclusion of “Secondary Suspects” has artificially inflated the DPA counts.

**Figure 2:**
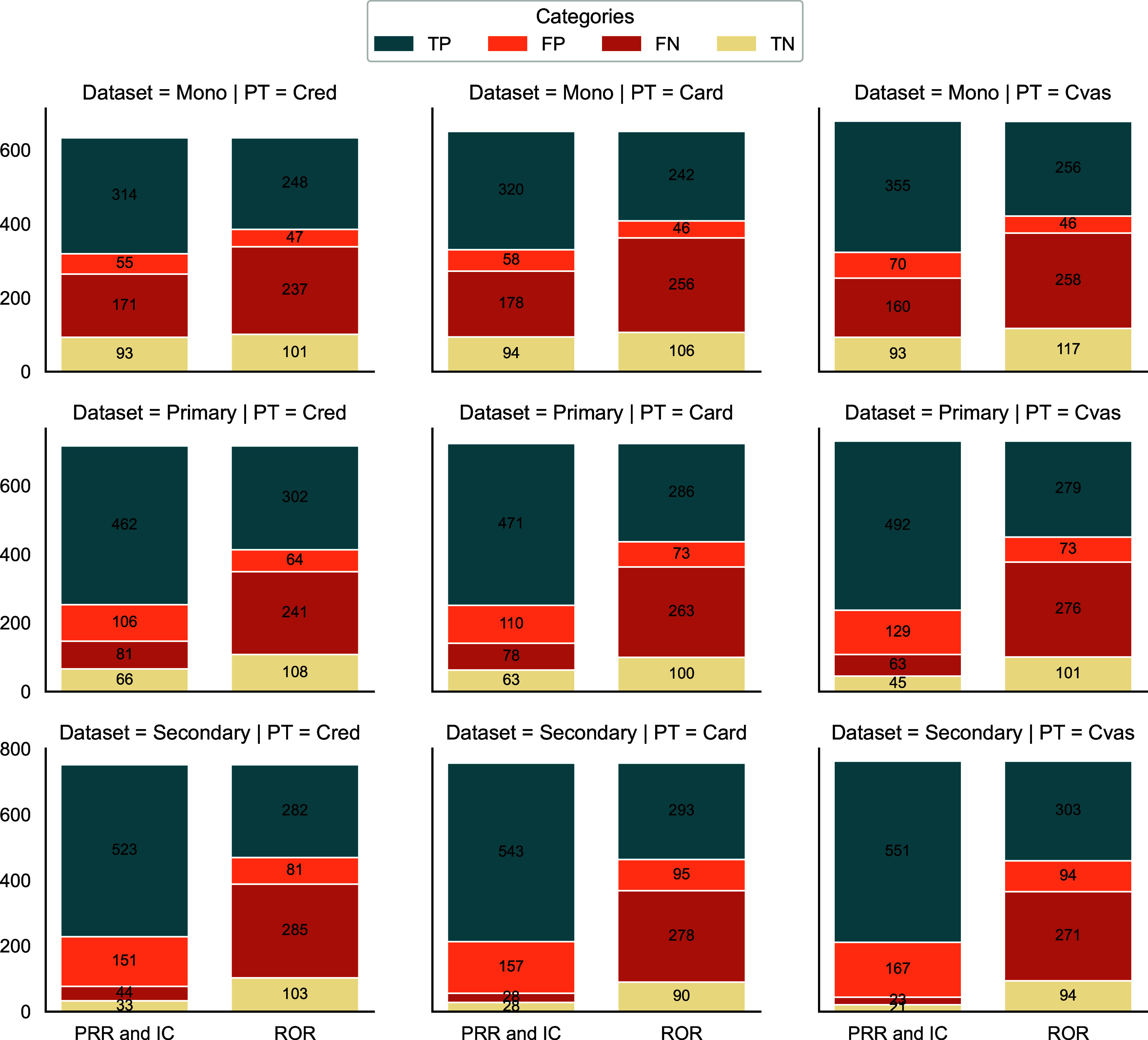
Nonstratified agreement with DICTrank for all CARBIDE variants. TP—true positive, FP—false positive, FN—false negative, and TN—true negative.

ROR-based assignments were more conservative (50–57% agreement), with fewer true positives and more false negatives. PRR- and IC-based assignments showed higher overall agreement (64–76%) and produced identical classification distributions due to the mathematical similarity of their mean values relative to the set thresholds (Supporting Information, Figure S1).

The choice of MedDRA PTs used to define cardiotoxic events had comparatively minor effects on the class distributions. With PRR and IC, broader term sets (Card, Cvas) resulted in more compounds being classified as cardiotoxic, while with ROR, the opposite trend was observed.

### 2.4 Model Performance Analysis

#### 2.4.1 Performance in Subpopulations

On the basis of the obtained results and performed statistical tests, we selected the Primary-Cred-PRR configuration as optimal, given its balanced predictive performance, good agreement with DICTrank, and conservative clinical scope.

Model performance (measured by ROC AUC, PRC AUC, and HarmRS) was generally comparable between types of descriptors, with XGBoost yielding slightly better and more stable performance across folds, particularly when combined with ECFP descriptors (Supporting Information, Figure S2). For these reasons, this combination was used throughout subsequent analyses.

To investigate whether the models learned meaningful interactions between demographics and molecular structures, we calculated performance metrics within each subpopulation and assessed how predicted class probabilities changed when specific demographic characteristics were present, using distribution means as a baseline (Figure 3). ROC AUC and PRC AUC values remained within a comparable range across demographic subgroups (0.55–0.63 and 0.58–0.72, respectively), while the Recall-Specificity ratio (RS ratio) varied substantially. When stratified by sex, we observed a lower recall (around 0.41) and increased specificity (around 0.72) for Males and more balanced results for Females (0.59 and 0.56, respectively). When stratified by age, we observed a transition from models with higher RS ratios in younger populations to those with lower ratios in older populations. The differences were the least pronounced in stratification by weight, with the highest-weight group having the lowest RS ratio.

**Figure 3:**
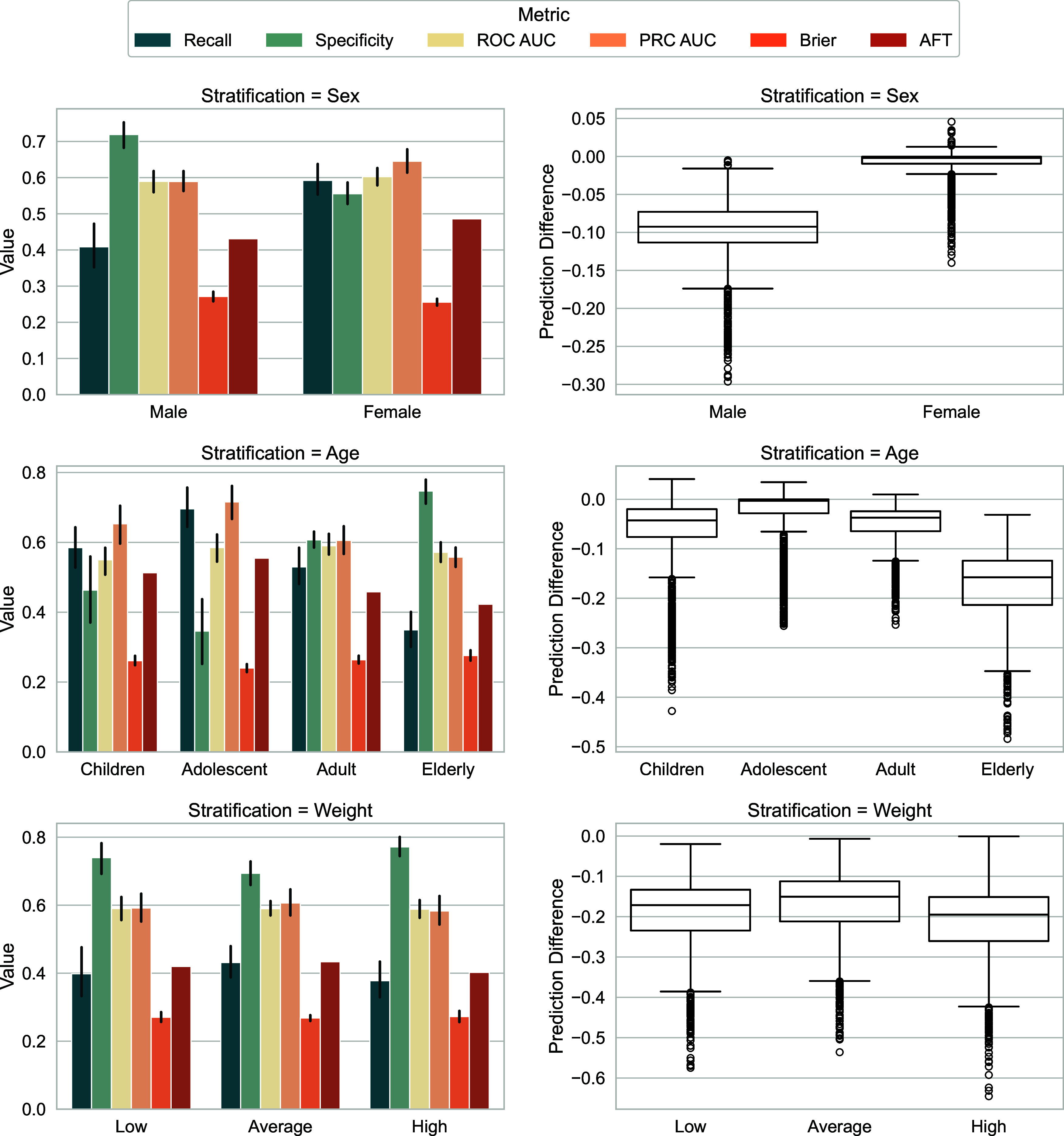
Performance of an XGBoost model combined with ECFP descriptors on the selected CARBIDE variant (left) and toxic class probability prediction change when given demographic factor was present (right). AFT—Adjusted Fraction of Toxic compounds.

As these trends appeared to reflect systematic relationships, we investigated the correlation between the RS ratio and the Adjusted Fraction of Toxic compounds (AFT), calculated as the sum of the label weights of toxic compounds divided by the total label weight sum. Our analysis revealed an almost perfectly linear relationship, suggesting that the models likely learned to predict encoded population-level statistics rather than capturing meaningful interactions between molecular and demographic features (Supporting Information, Figure S3 and Table S6).

### 2.5 Ablation Analysis

We conducted two complementary analyses to understand the role of demographic information in our models. First, we evaluated the importance of structural and demographic features through an ablation study comparing three scenarios: (a) structure-only prediction, where demographic features were set to zero; (b) demographics-only prediction, where structural descriptors were set to zero; and (c) a naïve baseline that predicts based on cardiotoxicity rates within demographic subgroups. Second, we investigated whether focusing on specific demographic factors during DPA-based label assignment might improve predictive performance. This was done by comparing our Combined-demographics approach against models trained on No-demographic and Single-demographic factors: sex-only, age-only, and weight-only data. The results of both analyses are presented in Figure 4, showing feature contribution comparisons (Panel A) and Single-demographic model performance (Panel B). The results of these analyses further confirm our initial hypotheses about the models’ use of demographic information. When provided with only demographic features, models essentially learn to reproduce cardiotoxicity rates within subpopulations and perform similarly to the naïve prevalence predictor. Structure-only models performed comparably to the combined approaches, suggesting that the addition of demographic features provides almost no benefit beyond what can be learned from molecular structure alone.

**Figure 4:**
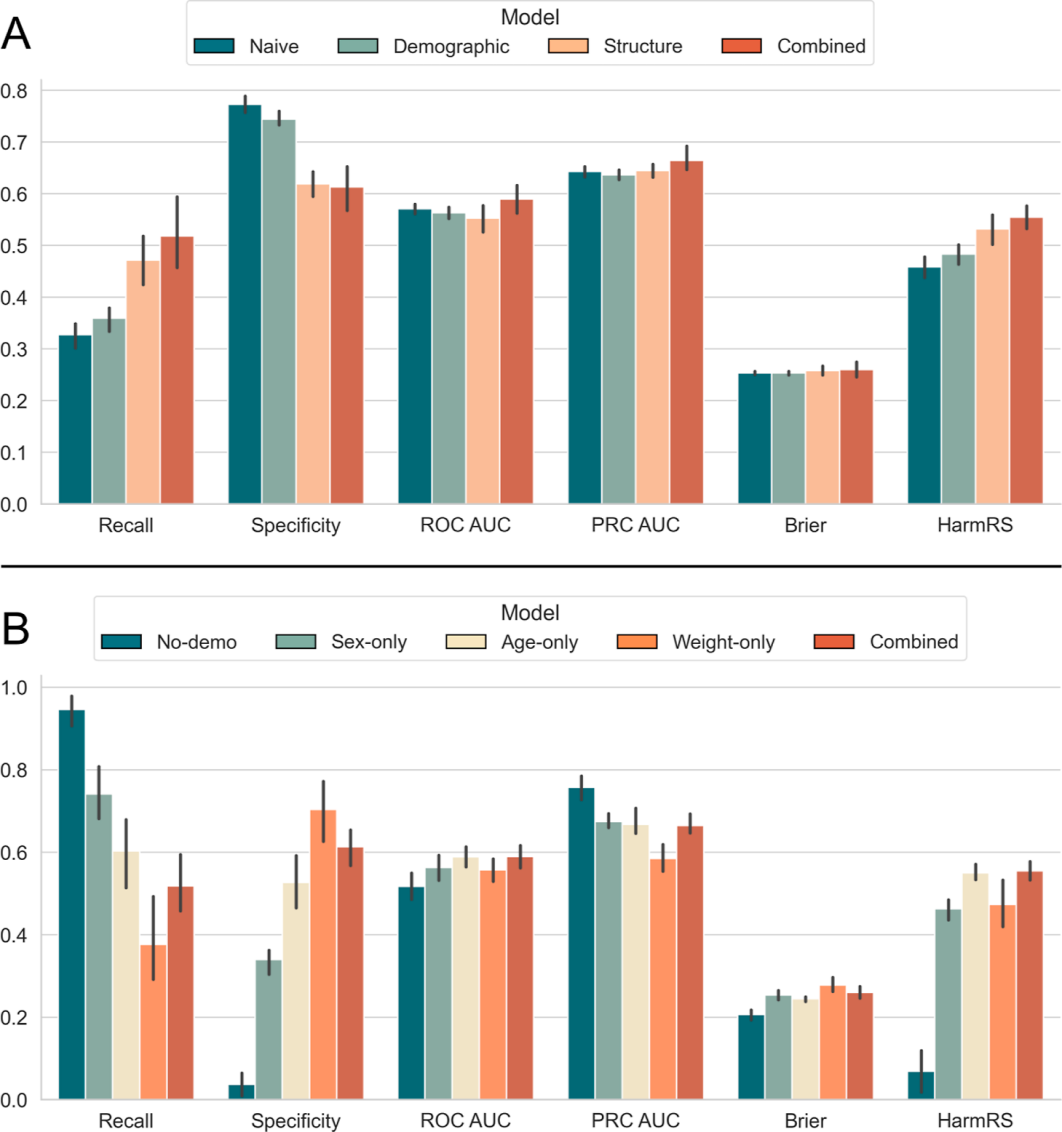
Ablation analysis results. (A) Performance comparison between different feature combinations: naïve prevalence predictor (baseline), demographics-only prediction, structure-only prediction, and the model combining both feature types. (B) Model performance when trained on different demographic subsets: No-demographic, Sex-only, Age-only, Weight-only, Combined-demographic.

Similarly, combining multiple stratification levels with the aim of allowing models to learn about inter-demographic interactions provides no measurable benefit. The No-demographic and Single-demographic models performed comparably to the combined model. The main differences were observed in the Recall-Specificity ratio, which again appears to be strongly influenced by the AFT, which in turn depends on data availability.

To verify that our observations were not artifacts of the splitting strategy, we analyzed the relationship between test-train set distances and model errors when using ECFP descriptors. For each prediction, we calculated the minimum Tanimoto distance to training set compounds and the corresponding absolute prediction error. The analysis yielded Pearson’s |*r*| < 0.1 across all models (Supporting Information, Figure S4 and Table S7).

## 3 Conclusions and Outlook

In this study, we systematically evaluated whether pharmacovigilance data could be used to develop demographically aware cardiotoxicity models. Through a comprehensive analysis of different CARBIDE variants regarding FAERS filtering criteria, cardiotoxicity definitions, and DPA metric choices, we found that this approach faces fundamental limitations.

While we identified the Primary-Cred-PRR as the optimal configuration, our analyses and ablation studies revealed that the models failed to learn meaningful structure-demographic interactions. Instead, they either learned population-level statistics or relied almost exclusively on molecular structures, with demographic information providing little additional predictive value.

The failure of trained models to learn meaningful interactions likely stems from two key factors. First, extracting clear structure-toxicity relationships from pharmacovigilance data via DPA methods is inherently difficult due to several limitations: inability to establish causality (DPA identifies associations rather than causes), systematic reporting biases, and the presence of duplicates.^40,41^ Second, the naïve prevalence predictor achieving comparable performance to trained ML models suggests the bottleneck lies in the data rather than the modeling methodology. A solution to this problem might be the use of more sophisticated data extraction approaches that could better account for the limitations of FAERS data, such as the use of deep learning for text mining or named entity recognition for the extraction of adverse events and active ingredients.^42^

However, a more promising direction would be to focus on approaches that rely on higher quality data sources or more relevant features. These include (a) integration of electronic health records with detailed patient histories and controlled interventions, (b) incorporation of mechanistic data like target binding profiles (e.g., cardiac ion channels or hormone receptors), genomic data, or molecular events related to known cardiotoxicity mechanisms, and (c) development of physiologically based models that explicitly account for demographic differences in pharmacokinetics and pharmacodynamics.

Ultimately, our results highlight the limitations of pharmacovigilance-based predictive modeling. This study provides a systematic evaluation of the challenges in demographic-aware drug safety modeling and identifies more promising directions for the development of effective personalized frameworks. Beyond these insights, CARBIDE itself represents a concrete contribution: an open, systematically constructed benchmarking resource that the community can build upon as better data sources and modeling strategies become available.

## 4 Materials and Methods

### 4.1 Pharmacovigilance Data Preparation

#### 4.1.1 Data Sourcing and Preparation

Pharmacovigilance data were retrieved from the FAERS database^20^ (from Q4 2012 to Q3 2024, where Q denotes calendar quarters). Individual files were merged, and fields that were not relevant to the study (such as duration of therapy, admission time, and formulation) were removed. To address known duplication issues in pharmacovigilance databases,^43^ we retained only the most recent case for each unique PRIMARYID. The drug descriptions were then mapped to active ingredients using a multistep approach, first applying synonym dictionaries and web-scraping heuristics (Supporting Information, Algorithm S1) and then expanding coverage through string similarity matching based on Damerau–Levenshtein distance (threshold: 0.85). This process resulted in a final mapping of approximately 311,000 drug descriptions to 8260 drug combinations, including 4333 unique compounds (Supporting Information, Section 3.1).

#### 4.1.2 Drug to SMILES Mapping

Drug names were mapped to SMILES strings using PubChem API^44^ and the NCI CADD Chemical Identifier Resolver.^45^ Missing entries were reviewed and manually added using the PubChem database when the structures were identifiable. We obtained SMILES strings for a total of 3759 compounds. The SMILES were standardized by removing stereochemistry information, neutralizing charges, salt stripping, and tautomer standardization using RDKit. Compounds were discarded if they met any of the following criteria: organometallic structure, molecular weight >800 Da, >80 heavy atoms, total polar surface area >250, or logP outside the [−5, 9] range. The final set subjected to analysis consisted of 2970 unique compounds.

#### 4.1.3 Demographic Data Processing

The FAERS database contains three demographic fields: sex, age, and weight. The sex field was used as is, without additional processing. Age and weight fields (values and units) were combined to express age in months and weight in kilograms.

Age was categorized into four groups following the FDA age classification: Children (0–12 years), Adolescents (12–21 years), Adults (21–65 years), and the Elderly (65–100 years). Although the FDA distinguishes neonates (birth to 1 month), infants (1 month to 2 years), and children (2–12 years) as separate subcategories, these were consolidated into a single Children group due to limited data availability in the younger age ranges. Entries with negative or implausible values (>100 years) were considered erroneous and encoded as “Unknown”. The proportion of missing and removed entries for each demographic field is given in Supporting Information, Table S8.

The weight was categorized into three groups (Low, Average, and High) relative to each demographic subpopulation. We calculated quantile thresholds (0.05, 0.33, 0.67, and 0.95) of weight distributions stratified by sex and age groups. For entries missing sex information, we applied age-specific quantiles. When the age group was unknown but the weight was available, we estimated the appropriate bin by comparing the weight to age-specific weight ranges (using the 0.1 and 0.9 quantiles). When the weight could belong to multiple age groups, we used the average of the corresponding quantile thresholds. In this way, the weight was categorized relative to demographic peers rather than absolute values (Supporting Information, Figure S5).

#### 4.1.4 FAERS Filtering

We investigated three base versions of CARBIDE that differed in filtering criteria. The first version (Mono) included only entries corresponding to monotherapy, i.e., a single drug in a record. The second version (Primary) expanded it by including only drugs labeled “Primary Suspect” in the Role Code field, regardless of potential polytherapy. The third version (Secondary) additionally included drugs labeled “Secondary Suspect”. Entries were removed if they contained more than 20 reported adverse drug reactions (all variants), more than one primary suspect (Primary), or more than 10 reported drugs (Secondary). This was done to exclude (a) entries for which establishing a drug-adverse outcome relationship was implausible and (b) entries likely submitted by drug manufacturers.

### 4.2 Label Assignment

Labels were assigned using DPA methods,^23^ which quantify the strength of associations between drugs and adverse events by comparing observed versus expected co-occurrences in a pharmacovigilance database.

#### 4.2.1 Cardiotoxicity Definition

We based our definitions on the MedDRA PTs.^22^ To investigate the influence of cardiotoxicity definition scope, we prepared three versions, each constructed by manually reviewing all terms within the relevant System Organ Class (SOC) and retaining only those pertinent to cardiotoxicity. In the “Cardiac Reduced” set (Cred, 341 terms), we included only terms from the “Cardiac disorders” SOC. This set was expanded to the “Cardiac Toxicity” (Card, 483 terms) set by adding terms from “General disorders” and “Investigations” SOCs. The last set, “Cardiovascular Toxicity” (Cvas, 848 terms), additionally included terms from the “Vascular disorders” SOC.

#### 4.2.2 DPA Metrics

We used three DPA metrics: PRR,^24^ ROR,^25^ and IC^26^—along with their CIs—to quantify the strength of putative cardiotoxicity signals.

PRR and ROR are widely used frequentist methods, whereas IC, derived from Bayesian modeling, provides a probabilistic perspective. The values and CIs were computed using an in-house Python implementation based on the equations described in the *pvda* R package^46^ (Supporting Information, eqs 1–12).

#### 4.2.3 Assignment Criteria

We classified the compounds into four cardiotoxicity risk classes based on the relative positions of the CIs and thresholds: High, Moderate, Low, and Minimal (Figure 5). Following recommendations from the literature,^23^ we used a threshold of 1 for PRR and ROR, 0 for IC, and removed entries with fewer than three co-occurrences of drug-adverse events from the analysis.

**Figure 5:**
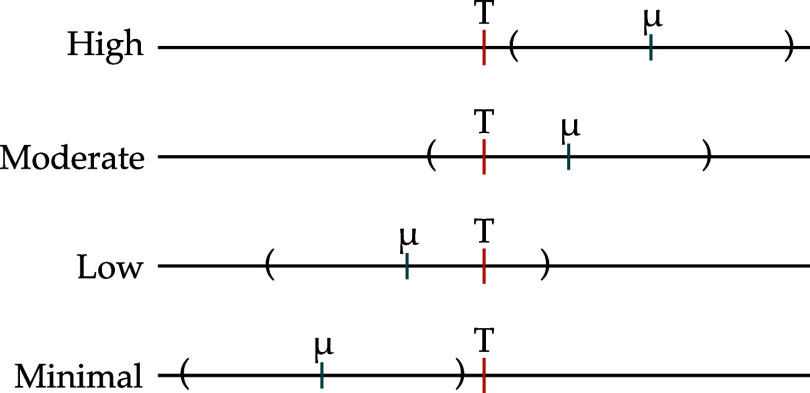
Assignment criteria for cardiotoxicity risk classes. Compounds are assigned to one of four classes (High, Moderate, Low, Minimal) based on the relative position of the calculated value (μ) and its CI (brackets) to the threshold (T) for a given DPA metric.

#### 4.2.4 Demographic Stratification

To capture demographic-specific signals, the DPA metrics were calculated independently within subgroups defined by sex, age, weight, and their combinations. We evaluated four stratification levels: no stratification (all entries pooled), single-factor (e.g., by sex: Male, Female, and Unknown), two-factor (e.g., sex × age), and full stratification (sex × age × weight). The results were then aggregated for downstream analysis.

#### 4.2.5 Label Confidence Scores

We assigned confidence scores to each label to quantitatively reflect the statistical certainty of our findings based on the amount and variability of available data (Supporting Information, Section 3.3). During further evaluations and model training, the labels were binarized, with High and Moderate classes being considered Toxic and Low and Minimal as Non-toxic. Following the analysis, the number of unique compounds decreased to 1986 due to an insufficient number of drug-adverse reaction pairs for some compounds.

### 4.3 Dataset Selection

We prepared multiple versions of the dataset that varied in filtering criteria, cardiotoxicity definitions, and DPA metrics used to obtain the labels. To select the most suitable configuration, we (a) compared various characteristics of the prepared dataset variants, (b) evaluated a range of classical ML models and descriptors, and (c) assessed class agreement and chemical domain overlap with DICTrank,^17^ an existing cardiotoxicity dataset based on FDA labeling documents. For point (a), we used labels obtained without stratification by demographic factors, as demographic stratification is not available in the DICTrank dataset.

### 4.4 Machine Learning Models

#### 4.4.1 Selected ML Approaches

For each CARBIDE version, we trained three types of classification models: LR^27^ and RF^28^ from the scikit-learn (v. 1.8.0) library,^48^ and the eXtreme gradient boosting (XGB) model from the xgboost (v. 3.1.3) library.^49^ These were selected on the basis of their support for sample weights and parallel computation.

#### 4.4.2 Demographic and Molecular Descriptors

Each model was combined with six types of descriptors: ECFP,^30^ MACCS,^31^ and Klekota & Roth^32^ fingerprints; CDDD^33^ and ChemBERTa^34^ embeddings; and RDKit descriptors^35^ (Supporting Information, Table S9). Demographic information was encoded as binary dummy vectors, with the Unknown category represented as zeros, and then concatenated with molecular descriptors. For molecular descriptors, we removed columns containing at least one undefined, infinite, or very large value (>1 × 10^12^). Features with zero or near-zero variance (<1 × 10^–3^) were identified and removed using the VarianceThreshold class available in scikit-learn. Highly correlated features (Pearson |*r*| > 0.9) were removed iteratively: at each step, the feature with the highest number of pairwise correlations exceeding the threshold was removed, until no correlated pairs remained. Continuous descriptors were scaled using the RobustScaler (0.05 and 0.95 quantiles) available in scikit-learn. All preprocessing steps were performed within the training folds to ensure that no information from the test data leaked into the training process.

#### 4.4.3 Data Splitting

To our knowledge, no external dataset with demographic-stratified cardiotoxicity labels exists. Direct comparison across CARBIDE variants is also challenging because of differences in labels and entry weights. We considered using only compounds with consistent labels across all variants, but the resulting subset (768 of 1986 compounds) was demographically unrepresentative and class imbalanced.

Instead, all unique molecules were first clustered using the Butina algorithm^36^ with ECFP fingerprints (radius = 2, nBits = 4096) and a Tanimoto distance threshold of 0.75, empirically selected as the largest value yielding approximately equal fold sizes. To provide a rigorous and realistic evaluation scenario (i.e., evaluation on novel compounds that will not necessarily be similar to known drugs), each cluster (N = 552) was assigned as a whole to one of the five folds (Supporting Information, Figures S6 and S7) using the GroupKFold class from the scikit-learn library and used for nested cross-validation.

#### 4.4.4 Model Evaluation

For each model–descriptor combination, we performed hyperparameter optimization using Optuna (version 4.6.0).^50^ Within each outer fold, the inner loop ran 64 Optuna trials, with each trial evaluated using 4-fold cross-validation. Models with the best scores were ensembled using a cross-fold aggregation approach, combining models trained on different fold combinations (e.g., folds 1–3 and folds 2–4) within a single Optuna trial. The ensembles were evaluated on the corresponding outer test fold with the process repeated for all five folds (Figure 6). A total of 2430 optimization runs were performed. The optimized parameters and their ranges are given in the Supporting Information, Table S10.

**Figure 6:**
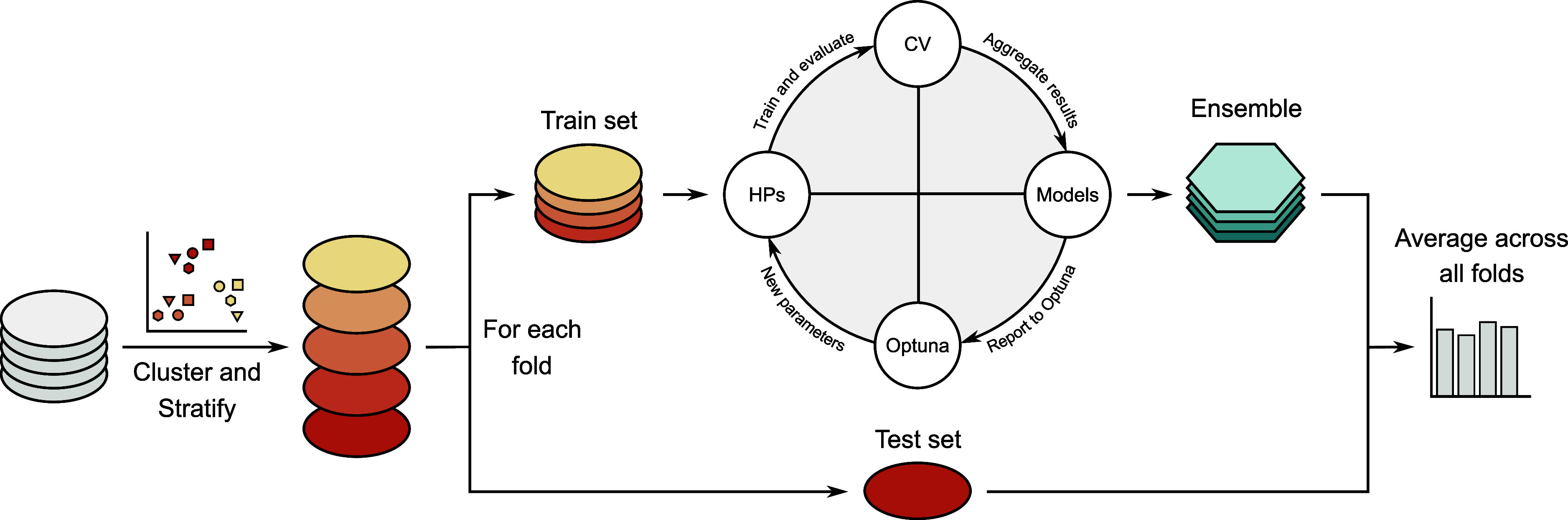
Nested cross-validation setup used for hyperparameter optimization and model evaluation. In each iteration, one outer fold served as the test set, while the remaining four folds were used for inner cross-validation (hyperparameter tuning) and ensemble training.

#### 4.4.5 CARBIDE Variant Comparison

For CARBIDE variant comparisons, models were evaluated only on entries with matching toxicity labels within each demographic subgroup, ensuring a fair comparison while maintaining adequate sample sizes. For example, consider the PRR metric, the Cred set, and an XGBoost model with MACCS descriptors. Two ensembles would be trained—one on the Primary version and the other on the Secondary—using folds 1–4. The fifth fold, unused during training or evaluation, would be filtered to identify entries with matching labels across both variants. Then, the ensembles would be evaluated on this subset. This process was repeated across all folds, models, and descriptors.

#### 4.4.6 Statistical Comparison

The metrics obtained in this way were then used to compare the CARBIDE variants using two-sided Wilcoxon signed-rank tests^37^ as implemented in SciPy (v. 1.17.0).^51^ For each comparison (e.g., Primary vs Secondary, PRR vs ROR, Cred vs Card), the matched pairs were defined by the unique combination of model type, descriptor, test fold, and the two design choices not evaluated at a given time (i.e., when filtering criteria were evaluated, the PTs and DPA metric type were included in pairing). HarmRS, ROC AUC, and PRC AUC were used as the comparison metrics. Effect sizes were quantified using the matched-pairs rank–biserial correlation^39^ implemented in statsmodels (v. 0.14.6)^52^, with |*r*_b_| < 0.1 interpreted as negligible, 0.1–0.3 as small, 0.3–0.5 as medium, and ≥0.5 as large effects.^53^ To control the family-wise error rate across the comparisons, the *p*-values were adjusted using the Holm step-down procedure.^38^ A significance level of α = 0.01 was used for all tests.

## Supplementary Material



## Data Availability

The CARBIDE dataset, the source code, and the Jupyter notebooks required to reproduce the results of this study are publicly available at https://github.com/M-Iwan/CARBIDE under the Creative Commons Attribution 4.0 International (CC-BY 4.0) license. All data and model artifacts, including raw FAERS data, associated mapping files, raw and aggregated results and analyses, and trained ML models, are archived at 10.5281/zenodo.18605831.

## Notes

Confidence score distributions, performance of trained models on the selected CARBIDE variant, relationship between RS ratio and adjusted fraction of toxic compounds, correlation between the minimum Tanimoto distance to training folds and absolute error, demographic-specific weight categorization, cross-validation distributions of minimum Tanimoto distances, UMAP projection of data folds, confusion matrix for DPA analysis, sigmoid function used for confidence scores, CARBIDE variants characteristics, details of statistical comparisons, model performance by filtering criteria, model performance by cardiotoxicity definition, model performance by DPA metric, class and label weights distributions, summary of missing and removed entries, overview of used molecular descriptors, hyperparameter optimization space, FAERS processing and drug mapping algorithm, disproportionality analysis equations (PRR, ROR, and IC with confidence intervals), label confidence score calculation procedure, and definitions of used performance metrics (PDF)
