# Supplementary material for: Beyond Molecular Structures: Investigating Demographic Factors in Drug-Induced Cardiotoxicity Prediction Models
Source: J Chem Inf Model. 2026 Jun 2;66(12):6962–71. doi: 10.1021/acs.jcim.6c00418 (PMC13292213; doi:10.1021/acs.jcim.6c00418)
Supplement: Supplementary file 1 [file ci6c00418_si_001.pdf]

SUPPORTING INFORMATION

Beyond Molecular Structures: Investigating Demographic Factors in Drug-Induced Cardiotoxicity Prediction Models

Mateusz Iwan[a,b,*], **Alessandra Roncaglioni**[b], **Francesca Grisoni**[a]

[a] Department of Biomedical Engineering, Eindhoven University of Technology, Institute for Complex Molecular Systems (ICMS), Eindhoven 5600 MB, The Netherlands
[b] Department of Environmental Health Sciences, Istituto di Ricerche Farmacologiche Mario Negri IRCCS, Milan 20156, Italy
[*] Corresponding author. Email: m.iwan@tue.nl, ORCID: 0000-0001-5151-4659

Table of Contents

1. **Supporting Figures**
   - **Figure S1:** Distributions of confidence scores used as sample weights for the investigated DPA metrics.
   - **Figure S2:** Performance metrics of the trained models on the selected CARBIDE variant.
   - **Figure S3:** Relationship between Recall-Specificity ratio and Adjusted Fraction of Toxic compounds.
   - **Figure S4:** Correlation between absolute error and minimum Tanimoto distance to training folds.
   - **Figure S5:** Demographic-specific weight categorization.
   - **Figure S6:** Cross-validation distributions of minimum Tanimoto distances to training folds.
   - **Figure S7:** UMAP projection of data folds.
   - **Figure S8:** Confusion matrix for DPA assignments.
   - **Figure S9:** Sigmoid function used for confidence scores.
2. **Supporting Tables**
   - **Table S1:** Composition and characteristics of CARBIDE variants.
   - **Table S2:** Results of statistical comparisons between CARBIDE variants.
   - **Table S3:** Impact of filtering criteria on model performance.
   - **Table S4:** Impact of cardiotoxicity definitions on model performance.
   - **Table S5:** Impact of DPA metric selection on model performance.
   - **Table S6:** Distribution of class and label weights.
   - **Table S7:** Correlation between absolute error and minimum Tanimoto distance to training folds.
   - **Table S8:** Summary of missing and removed entries by demographic subpopulations.
   - **Table S9:** Overview of the molecular descriptors used in the study.
   - **Table S10:** Hyperparameter optimization search space.
3. **Methodological Details**
   - **Section 3.1:** FAERS processing and mapping algorithm.
   - **Section 3.2:** Disproportionality Analysis (DPA) methodology.
   - **Section 3.3:** Label assignment.
   - **Section 3.4:** Evaluation metrics.
4. **Bibliography**

1. Supplementary Figures

1.1. Confidence Scores for the three investigated DPA metrics

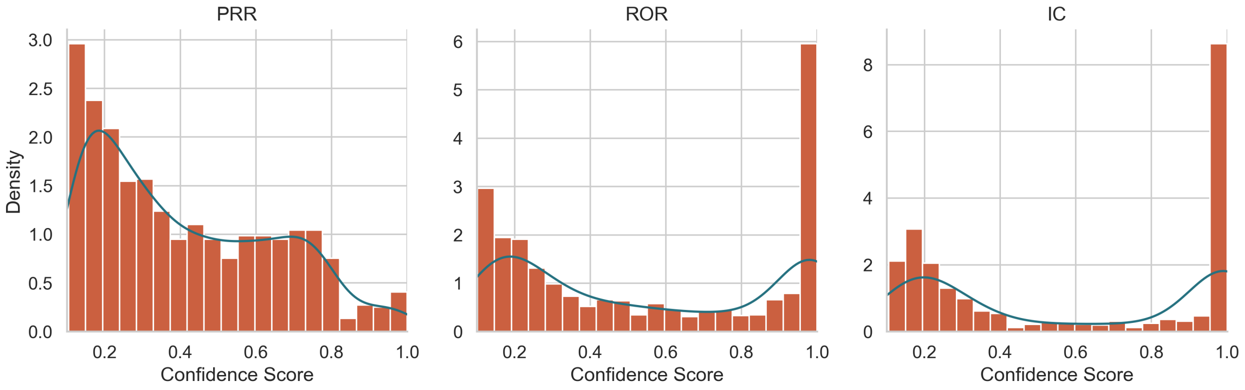

Figure S1. Histograms showing the distribution of confidence scores for cardiotoxicity classifications in the Primary dataset using the Cred PTs. While the binarized labels for PRR and IC are identical, the calculated confidence intervals and in turn confidence scores differ. PRR - Proportional Reporting Ratio, ROR - Reporting Odds Ratio, IC - Information Component.

1.2. Performance metrics of the trained models on the selected CARBIDE variant

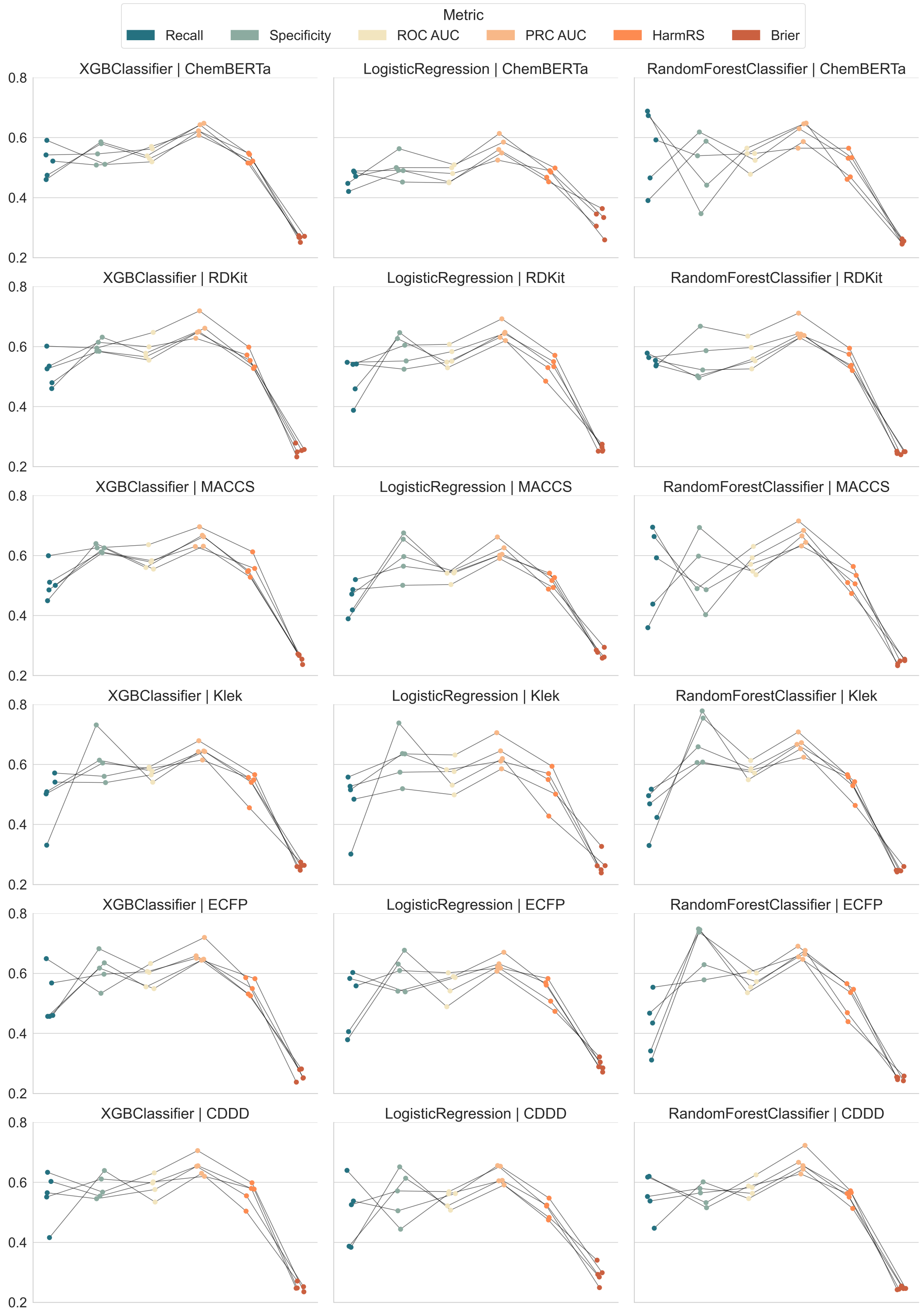

Figure S2. Performance statistics on the selected CARBIDE variant by model and descriptor types. Each dot corresponds to an evaluation on an outer test fold. ROC AUC - Receiver Operating Characteristic—Area Under Curve, PRC AUC - Precision Recall Curve—Area Under Curve, HarmRS - Harmonic mean of Recall and Specificity.

1.3. Relationship between Recall-Specificity ratio and Adjusted Fraction of Toxic compounds

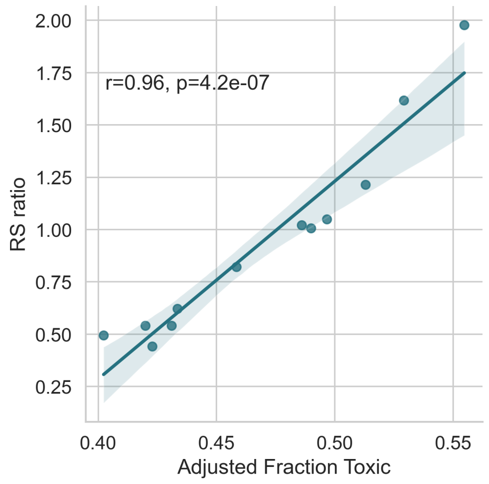

Figure S3. Linear regression analysis of Recall-Specificity ratio (RS ratio) and Adjusted Fraction of Toxic compounds (AFT). Each point represents a demographic subpopulation, showing the relationship between the weighted proportion of toxic compounds and the resulting model behavior.

1.4. Correlation between absolute error and minimum Tanimoto distance to training folds

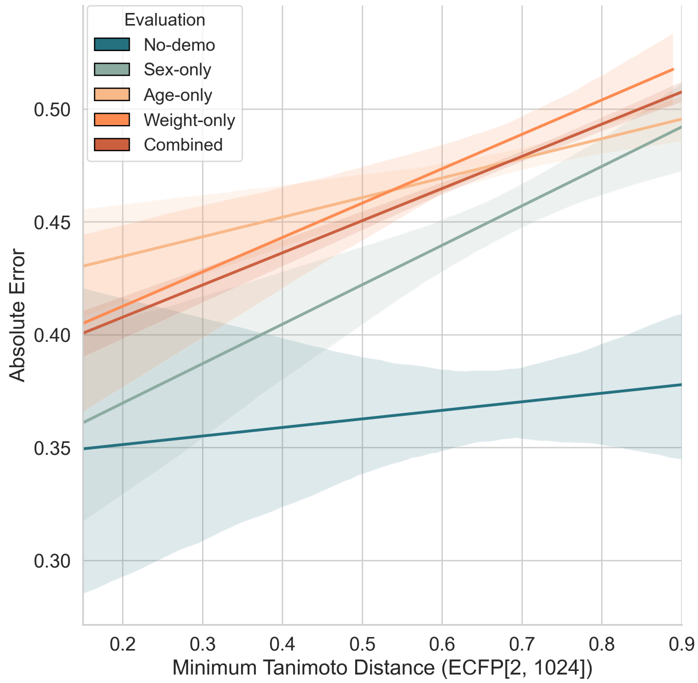

Figure S4. Correlation between absolute error and minimum Tanimoto distance to training folds. Absolute Error was calculated as an absolute difference between toxicity label and predicted probability. Extended-Connectivity Fingerprints (radius = 2, nBits = 1024) were used for distance calculation.

1.5. Demographic-specific weight categorization

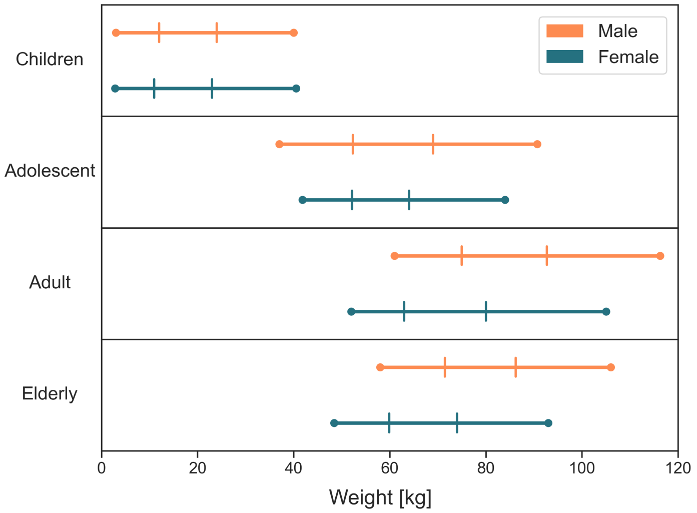

Figure S5. Weight quantile thresholds for demographic-specific categorization. The figure illustrates how weight categories (Low, Average, High) were assigned relative to demographic peers rather than using absolute cutoffs. Vertical lines represent quantile-based breakpoints (0.33 and 0.67) used to define weight categories within each sex × age subgroup.

1.6. Cross-validation fold distribution and chemical space coverage

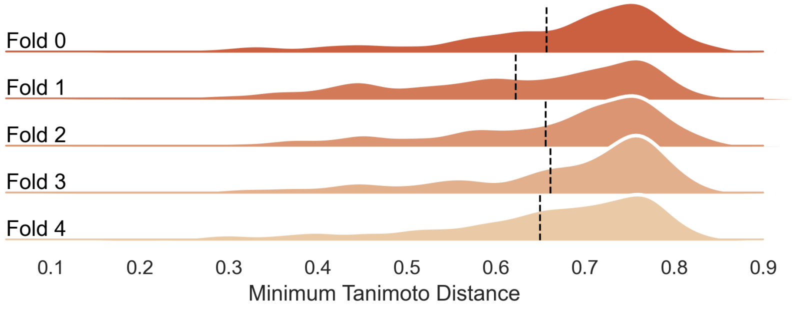

Figure S6. Distribution of minimum inter-fold Tanimoto distances following Butina clustering. Kernel Density Estimate (KDE) plots show the distribution of minimum Tanimoto distances between molecules in each test fold and their nearest neighbors in the corresponding training folds. Clustering was performed using ECFP fingerprints (radius = 2, nBits = 4096) with a distance threshold of 0.75.

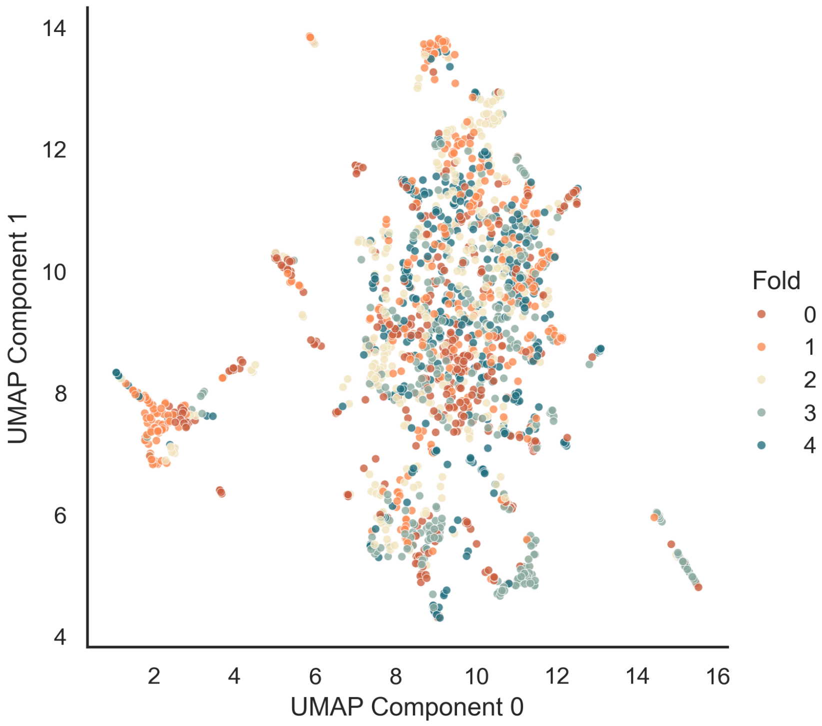

Figure S7. UMAP projection of chemical space distribution across five data folds, with each point representing a molecule colored by its assigned fold.

2. Supporting Tables

2.1. Composition of CARBIDE variants

Table S1. Characteristics of different CARBIDE variants. Dataset - filtering criteria, PTs - MedDRA Preferred Terms used to describe cardiotoxicity, DPA Metric - Disproportionality Analysis Metric, ConfScore - Confidence Scores (used as sample weights during model training), EpS - Entries per SMILES.

Dataset	PTs	DPA Metric	#Entries	#SMILES	%Toxic	ConfScore (Tox)	ConfScore (Non-Tox)	EpS
Mono	Cred	PRR	14997	958	57.42	21 ± 17	40 ± 29	16 ± 13
		ROR	14978	958	56.48	19 ± 14	41 ± 30	16 ± 13
		IC	14997	958	57.42	29 ± 23	23 ± 16	16 ± 13
	Card	PRR	16343	986	57.70	22 ± 17	40 ± 29	17 ± 13
		ROR	16319	986	56.69	19 ± 14	41 ± 30	17 ± 13
		IC	16343	986	57.71	29 ± 23	24 ± 16	17 ± 13
	Cvas	PRR	19595	1063	58.88	24 ± 20	42 ± 30	18 ± 14
		ROR	19551	1062	57.52	20 ± 16	44 ± 31	18 ± 14
		IC	19595	1063	58.88	30 ± 25	25 ± 17	18 ± 14
Primary	Cred	PRR	26273	1156	57.78	28 ± 22	42 ± 29	23 ± 16
		ROR	26259	1156	54.56	24 ± 20	46 ± 31	23 ± 16
		IC	26273	1156	57.79	38 ± 30	25 ± 16	23 ± 16
	Card	PRR	28131	1191	58.75	28 ± 23	43 ± 29	24 ± 16
		ROR	28109	1191	55.33	25 ± 20	47 ± 32	24 ± 16
		IC	28131	1191	58.75	38 ± 30	26 ± 17	24 ± 16
	Cvas	PRR	32152	1249	58.36	32 ± 25	45 ± 30	26 ± 17
		ROR	32110	1248	54.23	27 ± 23	49 ± 32	26 ± 17
		IC	32152	1249	58.37	41 ± 31	27 ± 19	26 ± 17
Secondary	Cred	PRR	37571	1786	61.52	29 ± 22	45 ± 29	21 ± 16
		ROR	37504	1784	56.33	26 ± 21	51 ± 32	21 ± 16
		IC	37571	1786	61.52	42 ± 31	26 ± 17	21 ± 16
	Card	PRR	39815	1839	61.91	30 ± 23	45 ± 30	22 ± 17
		ROR	39720	1837	56.53	26 ± 22	51 ± 32	22 ± 17
		IC	39815	1839	61.91	43 ± 32	27 ± 17	22 ± 17
	Cvas	PRR	45683	1986	60.96	34 ± 26	46 ± 30	23 ± 17
		ROR	45526	1982	54.88	29 ± 24	53 ± 33	23 ± 17
		IC	45683	1986	60.97	45 ± 33	28 ± 19	23 ± 17

2.2. Results of statistical comparison between CARBIDE variants

Table S2. Results of statistical comparison between CARBIDE variants. ROC AUC - Receiver Operating Characteristic—Area Under Curve, PRC AUC - Precision Recall Curve—Area Under Curve, HarmRS - Harmonic mean of Recall and Specificity.

Null Hypothesis	$Wilcoxon_{stat}$	P_{value}	r_b	Effect size	$P_{value,adj.}$	Conclusion
			HarmRS			
PRR ∼ IC	21903	2.7e-101	0.87	Large	7.4e-100	PRR > IC
ROR ∼ IC	28277	1.4e-92	0.83	Large	3.6e-91	ROR > IC
Primary ∼ Secondary	67883	2.1e-47	0.59	Large	5.1e-46	Primary > Secondary
Mono ∼ Secondary	93335	1.9e-26	0.43	Medium	4.1e-25	Mono > Secondary
Cred ∼ Cvas	119088	1.2e-11	0.28	Small	2.5e-10	Cred > Cvas
PRR ∼ ROR	136318	2.8e-05	0.17	Small	0.00048	PRR > ROR
Cred ∼ Card	137797	9.1e-05	0.16	Small	0.0013	Cred > Card
Card ∼ Cvas	141666	0.00071	0.14	Small	0.0078	Card > Cvas
Mono ∼ Primary	141966	0.00083	-0.14	Small	0.0083	Mono < Primary
			ROC AUC			
Mono ∼ Primary	92486	7.4e-27	-0.44	Medium	1.7e-25	Mono < Primary
Mono ∼ Secondary	113310	2.1e-14	-0.31	Medium	4.4e-13	Mono < Secondary
Cred ∼ Cvas	139191	0.00017	0.15	Small	0.0022	Cred > Cvas
Card ∼ Cvas	138857	0.00017	0.15	Small	0.0022	Card > Cvas
Primary ∼ Secondary	142085	0.00089	0.14	Small	0.0083	Primary > Secondary
PRR ∼ ROR	154006	0.13	0.062	Negligible	0.75	PRR ∼ ROR
PRR ∼ IC	155448	0.19	0.054	Negligible	0.94	PRR ∼ IC
Cred ∼ Card	158250	0.37	0.036	Negligible	1.0	Cred ∼ Card
ROR ∼ IC	162940	0.85	-0.0078	Negligible	1.0	ROR ∼ IC
			PRC AUC			
Mono ∼ Primary	90992	4.1e-28	-0.45	Medium	9.7e-27	Mono < Primary
Primary ∼ Secondary	123799	1.3e-09	0.25	Small	2.4e-08	Primary > Secondary
Mono ∼ Secondary	128540	8.4e-08	-0.22	Small	1.5e-06	Mono < Secondary
Card ∼ Cvas	136324	2.8e-05	0.17	Small	0.00048	Card > Cvas
Cred ∼ Cvas	136730	3.7e-05	0.17	Small	0.00055	Cred > Cvas
PRR ∼ IC	150577	0.040	0.083	Negligible	0.32	PRR ∼ IC
ROR ∼ IC	151840	0.072	0.073	Negligible	0.50	ROR ∼ IC
Cred ∼ Card	161522	0.69	0.017	Negligible	1.0	Cred ∼ Card
PRR ∼ ROR	161847	0.72	0.015	Negligible	1.0	PRR ∼ ROR

2.3. Impact of filtering criteria on model performance

Table S3. Impact of filtering criteria on model performance. Dataset - FAERS filtering criteria, ROC AUC - Receiver Operating Characteristic—Area Under Curve, PRC AUC - Precision Recall Curve—Area Under Curve, HarmRS - Harmonic mean of Recall and Specificity, ECE - Expected Calibration Error, LR - Logistic Regression, RF - Random Forest, XGB - eXtreme Gradient Boosting.

Dataset	Model	Descriptors	Recall	Specificity	ROC AUC	PRC AUC	Brier	ECE	HarmRS
Mono	LR	CDDD	54 ± 10	53 ± 9	55 ± 5	66 ± 5	31 ± 4	23 ± 7	52 ± 4
		ChemBERTa	52 ± 10	52 ± 10	53 ± 8	64 ± 7	31 ± 4	23 ± 7	51 ± 5
		Klek	55 ± 10	55 ± 11	58 ± 5	68 ± 4	28 ± 3	18 ± 6	53 ± 5
		MACCS	55 ± 14	49 ± 16	53 ± 4	66 ± 5	28 ± 2	18 ± 4	48 ± 5
		ECFP	55 ± 11	53 ± 10	56 ± 7	66 ± 6	29 ± 4	21 ± 7	53 ± 6
		RDKit	56 ± 11	51 ± 12	56 ± 7	68 ± 5	28 ± 3	19 ± 4	51 ± 6
	RF	CDDD	64 ± 11	50 ± 12	61 ± 6	71 ± 6	24 ± 1	10 ± 4	54 ± 6
		ChemBERTa	62 ± 11	47 ± 10	57 ± 4	68 ± 4	24 ± 1	10 ± 4	52 ± 4
		Klek	49 ± 17	63 ± 18	59 ± 4	70 ± 5	25 ± 2	13 ± 6	50 ± 6
		MACCS	61 ± 11	53 ± 9	61 ± 5	72 ± 4	24 ± 1	10 ± 4	55 ± 5
		ECFP	49 ± 17	64 ± 17	60 ± 6	72 ± 5	25 ± 2	13 ± 5	51 ± 6
		RDKit	62 ± 11	51 ± 10	60 ± 6	70 ± 5	24 ± 1	10 ± 4	54 ± 5
	XGB	CDDD	64 ± 12	49 ± 13	61 ± 5	71 ± 4	24 ± 2	10 ± 4	53 ± 7
		ChemBERTa	62 ± 11	48 ± 12	58 ± 4	69 ± 4	25 ± 1	12 ± 4	52 ± 6
		Klek	60 ± 13	54 ± 15	60 ± 4	71 ± 4	25 ± 2	13 ± 4	54 ± 6
		MACCS	62 ± 12	53 ± 13	61 ± 4	72 ± 4	25 ± 2	11 ± 4	55 ± 5
		ECFP	60 ± 13	54 ± 12	60 ± 6	70 ± 5	25 ± 3	13 ± 5	54 ± 6
		RDKit	63 ± 12	52 ± 14	61 ± 4	72 ± 4	25 ± 2	12 ± 4	54 ± 5
Primary	LR	CDDD	57 ± 10	53 ± 9	57 ± 6	68 ± 5	29 ± 4	20 ± 5	54 ± 5
		ChemBERTa	52 ± 10	48 ± 8	50 ± 6	62 ± 6	31 ± 4	23 ± 6	49 ± 4
		Klek	57 ± 12	53 ± 12	57 ± 6	68 ± 6	28 ± 4	19 ± 5	53 ± 5
		MACCS	55 ± 17	52 ± 18	56 ± 3	68 ± 5	27 ± 2	16 ± 3	48 ± 6
		ECFP	58 ± 13	54 ± 10	58 ± 8	68 ± 7	28 ± 4	18 ± 6	54 ± 6
		RDKit	58 ± 15	53 ± 17	59 ± 6	70 ± 6	26 ± 2	15 ± 4	51 ± 7
	RF	CDDD	67 ± 13	51 ± 12	64 ± 5	74 ± 5	23 ± 1	10 ± 4	55 ± 6
		ChemBERTa	63 ± 15	46 ± 14	58 ± 5	69 ± 6	24 ± 1	9 ± 4	49 ± 7
		Klek	57 ± 19	57 ± 21	61 ± 4	72 ± 5	24 ± 2	11 ± 4	50 ± 8
		MACCS	64 ± 13	52 ± 12	63 ± 5	73 ± 5	23 ± 1	10 ± 4	55 ± 6
		ECFP	57 ± 18	60 ± 18	62 ± 5	73 ± 5	24 ± 2	11 ± 4	53 ± 7
		RDKit	66 ± 11	52 ± 11	63 ± 6	72 ± 6	23 ± 1	10 ± 3	56 ± 5
	XGB	CDDD	65 ± 13	54 ± 13	63 ± 4	73 ± 4	24 ± 1	10 ± 3	56 ± 5
		ChemBERTa	62 ± 12	49 ± 14	59 ± 4	71 ± 4	25 ± 1	11 ± 3	52 ± 7
		Klek	60 ± 15	55 ± 15	61 ± 4	72 ± 4	25 ± 2	12 ± 4	54 ± 6
		MACCS	61 ± 13	58 ± 14	63 ± 3	74 ± 4	24 ± 2	11 ± 3	57 ± 5
		ECFP	62 ± 14	55 ± 14	62 ± 6	73 ± 6	24 ± 2	11 ± 4	55 ± 6
		RDKit	64 ± 13	53 ± 13	63 ± 5	74 ± 5	24 ± 2	11 ± 4	56 ± 6
Secondary	LR	CDDD	59 ± 12	48 ± 12	55 ± 6	67 ± 6	28 ± 4	18 ± 6	50 ± 6
		ChemBERTa	56 ± 11	47 ± 9	52 ± 7	64 ± 6	30 ± 4	22 ± 6	49 ± 5
		Klek	61 ± 10	51 ± 10	59 ± 6	68 ± 6	27 ± 3	16 ± 6	54 ± 5
		MACCS	60 ± 21	47 ± 23	56 ± 3	68 ± 6	26 ± 2	14 ± 3	44 ± 11
		ECFP	60 ± 10	50 ± 9	57 ± 7	67 ± 7	27 ± 4	17 ± 6	53 ± 6
		RDKit	62 ± 19	50 ± 22	60 ± 4	70 ± 6	25 ± 2	13 ± 3	47 ± 10
	RF	CDDD	74 ± 16	39 ± 19	62 ± 6	72 ± 6	23 ± 1	8 ± 4	46 ± 14
		ChemBERTa	69 ± 18	34 ± 17	56 ± 6	68 ± 6	24 ± 1	8 ± 4	40 ± 13
		Klek	64 ± 20	48 ± 23	62 ± 3	72 ± 5	24 ± 1	9 ± 3	46 ± 13
		MACCS	68 ± 15	46 ± 15	62 ± 4	72 ± 5	23 ± 1	8 ± 4	51 ± 8
		ECFP	64 ± 20	49 ± 23	62 ± 5	72 ± 5	23 ± 2	9 ± 3	47 ± 12
		RDKit	72 ± 15	42 ± 16	61 ± 6	71 ± 7	23 ± 1	9 ± 3	49 ± 11
	XGB	CDDD	68 ± 15	45 ± 15	62 ± 4	71 ± 5	24 ± 1	9 ± 3	50 ± 8
		ChemBERTa	67 ± 14	41 ± 16	57 ± 4	69 ± 5	25 ± 1	11 ± 3	46 ± 10
		Klek	66 ± 16	48 ± 17	62 ± 3	72 ± 5	24 ± 1	10 ± 3	51 ± 9
		MACCS	65 ± 16	50 ± 18	62 ± 3	72 ± 4	24 ± 1	10 ± 3	51 ± 8
		ECFP	65 ± 16	48 ± 16	61 ± 7	71 ± 7	24 ± 3	12 ± 4	51 ± 9
		RDKit	68 ± 15	47 ± 16	63 ± 6	73 ± 6	24 ± 2	10 ± 3	52 ± 10

2.4. Impact of cardiotoxicity definition on model performance

Table S4. Impact of cardiotoxicity definition on model performance. PTs - MedDRA Preferred Terms used to denote cardiotoxicity,ROC AUC - Receiver Operating Characteristic—Area Under Curve, PRC AUC - Precision Recall Curve—Area Under Curve, HarmRS - Harmonic mean of Recall and Specificity, ECE - Expected Calibration Error, LR - Logistic Regression, RF - Random Forest, XGB - eXtreme Gradient Boosting.

PTs	Model	Descriptors	Recall	Specificity	ROC AUC	PRC AUC	Brier	ECE	HarmRS
Cred	LR	CDDD	54 ± 10	52 ± 10	54 ± 4	65 ± 5	30 ± 4	21 ± 6	51 ± 4
		ChemBERTa	53 ± 9	50 ± 8	52 ± 6	63 ± 6	31 ± 4	23 ± 6	50 ± 4
		Klek	55 ± 10	56 ± 11	57 ± 5	67 ± 5	28 ± 3	17 ± 6	54 ± 5
		MACCS	56 ± 17	51 ± 18	56 ± 3	66 ± 5	27 ± 2	16 ± 3	48 ± 7
		ECFP	54 ± 9	54 ± 9	56 ± 6	66 ± 6	29 ± 3	20 ± 5	53 ± 5
		RDKit	56 ± 15	53 ± 15	58 ± 5	68 ± 4	27 ± 2	15 ± 4	51 ± 6
	RF	CDDD	64 ± 13	49 ± 14	61 ± 4	71 ± 5	24 ± 1	9 ± 4	53 ± 9
		ChemBERTa	63 ± 14	45 ± 14	57 ± 4	67 ± 5	24 ± 1	8 ± 4	49 ± 9
		Klek	55 ± 20	58 ± 21	60 ± 3	70 ± 5	25 ± 2	11 ± 5	49 ± 9
		MACCS	61 ± 13	52 ± 12	60 ± 4	71 ± 4	24 ± 1	9 ± 4	54 ± 6
		ECFP	53 ± 20	60 ± 21	60 ± 4	70 ± 5	25 ± 2	11 ± 5	49 ± 8
		RDKit	63 ± 12	49 ± 12	60 ± 5	70 ± 5	24 ± 1	9 ± 3	53 ± 7
	XGB	CDDD	63 ± 12	51 ± 13	61 ± 4	70 ± 4	24 ± 1	10 ± 4	54 ± 6
		ChemBERTa	61 ± 12	49 ± 12	58 ± 4	68 ± 4	25 ± 1	12 ± 3	52 ± 6
		Klek	60 ± 15	54 ± 15	61 ± 4	70 ± 5	25 ± 2	12 ± 4	53 ± 6
		MACCS	60 ± 13	55 ± 15	61 ± 3	71 ± 4	25 ± 1	11 ± 3	54 ± 6
		ECFP	59 ± 13	55 ± 13	61 ± 5	70 ± 5	25 ± 2	12 ± 4	54 ± 5
		RDKit	62 ± 12	53 ± 13	62 ± 4	71 ± 5	25 ± 2	11 ± 4	55 ± 5
Card	LR	CDDD	55 ± 11	52 ± 9	55 ± 4	65 ± 5	30 ± 4	20 ± 6	52 ± 4
		ChemBERTa	53 ± 10	49 ± 8	52 ± 6	62 ± 6	31 ± 4	22 ± 6	50 ± 4
		Klek	56 ± 10	54 ± 11	58 ± 5	67 ± 5	27 ± 3	17 ± 5	54 ± 4
		MACCS	57 ± 17	51 ± 18	56 ± 3	67 ± 5	27 ± 2	15 ± 3	48 ± 7
		ECFP	55 ± 12	55 ± 10	57 ± 6	66 ± 6	28 ± 3	17 ± 6	53 ± 5
		RDKit	57 ± 15	52 ± 16	57 ± 5	68 ± 5	27 ± 2	16 ± 5	50 ± 6
	RF	CDDD	65 ± 15	47 ± 16	61 ± 5	71 ± 5	24 ± 1	8 ± 4	51 ± 10
		ChemBERTa	64 ± 16	43 ± 15	56 ± 5	67 ± 5	24 ± 1	8 ± 4	47 ± 10
		Klek	56 ± 20	56 ± 21	60 ± 4	70 ± 5	24 ± 2	11 ± 5	49 ± 9
		MACCS	62 ± 13	50 ± 12	60 ± 4	70 ± 4	24 ± 1	8 ± 4	53 ± 7
		ECFP	57 ± 19	55 ± 21	60 ± 4	70 ± 5	24 ± 2	10 ± 5	49 ± 9
		RDKit	64 ± 14	48 ± 13	60 ± 5	69 ± 5	24 ± 1	8 ± 4	52 ± 7
	XGB	CDDD	64 ± 13	50 ± 13	61 ± 4	70 ± 4	24 ± 1	10 ± 3	53 ± 6
		ChemBERTa	63 ± 12	47 ± 13	58 ± 3	68 ± 5	25 ± 1	11 ± 3	51 ± 7
		Klek	61 ± 14	54 ± 15	61 ± 3	70 ± 5	25 ± 2	12 ± 4	54 ± 6
		MACCS	61 ± 13	54 ± 14	61 ± 3	71 ± 4	25 ± 1	11 ± 3	54 ± 6
		ECFP	60 ± 15	53 ± 14	60 ± 5	70 ± 5	25 ± 2	12 ± 4	53 ± 6
		RDKit	63 ± 13	52 ± 13	61 ± 4	71 ± 5	24 ± 2	11 ± 3	54 ± 6
Cvas	LR	CDDD	56 ± 10	52 ± 9	56 ± 4	66 ± 4	29 ± 3	20 ± 6	52 ± 3
		ChemBERTa	54 ± 9	49 ± 9	53 ± 4	63 ± 6	30 ± 3	21 ± 5	50 ± 4
		Klek	57 ± 10	53 ± 10	57 ± 4	66 ± 5	28 ± 3	18 ± 5	53 ± 4
		MACCS	55 ± 18	51 ± 19	55 ± 4	65 ± 6	27 ± 2	15 ± 4	47 ± 6
		ECFP	58 ± 11	52 ± 9	56 ± 7	66 ± 6	29 ± 4	19 ± 6	53 ± 6
		RDKit	56 ± 16	52 ± 16	57 ± 5	67 ± 6	27 ± 3	16 ± 5	49 ± 7
	RF	CDDD	69 ± 14	44 ± 16	61 ± 5	70 ± 5	24 ± 1	8 ± 4	49 ± 11
		ChemBERTa	66 ± 14	42 ± 14	57 ± 3	67 ± 5	24 ± 1	8 ± 4	47 ± 9
		Klek	54 ± 20	56 ± 21	59 ± 4	69 ± 5	25 ± 2	11 ± 4	48 ± 8
		MACCS	64 ± 14	49 ± 13	61 ± 4	71 ± 4	24 ± 1	8 ± 4	52 ± 7
		ECFP	54 ± 19	58 ± 20	60 ± 5	70 ± 5	25 ± 2	11 ± 4	50 ± 8
		RDKit	67 ± 13	46 ± 13	60 ± 4	69 ± 5	24 ± 1	8 ± 3	52 ± 7
	XGB	CDDD	64 ± 13	48 ± 14	60 ± 3	69 ± 4	25 ± 1	10 ± 3	52 ± 7
		ChemBERTa	63 ± 12	46 ± 14	57 ± 3	68 ± 5	25 ± 1	12 ± 3	50 ± 6
		Klek	59 ± 15	53 ± 15	60 ± 3	69 ± 4	25 ± 2	12 ± 4	52 ± 6
		MACCS	61 ± 14	53 ± 15	61 ± 3	71 ± 4	25 ± 1	12 ± 3	53 ± 6
		ECFP	60 ± 15	53 ± 13	60 ± 6	69 ± 5	25 ± 2	12 ± 4	53 ± 6
		RDKit	63 ± 13	50 ± 14	60 ± 4	70 ± 4	25 ± 2	11 ± 3	52 ± 6

2.5. Impact of DPA metric on model performance

Table S5. Impact of DPA metric on model performance. DPA metric - Disproportionality Analysis metric used to calculate cardiotoxicity labels, ROC AUC - Receiver Operating Characteristic—Area Under Curve, PRC AUC - Precision Recall Curve—Area Under Curve, HarmRS - Harmonic mean of Recall and Specificity, ECE - Expected Calibration Error, LR - Logistic Regression, RF - Random Forest, XGB - eXtreme Gradient Boosting.

DPA metric	Model	Descriptors	Recall	Specificity	ROC AUC	PRC AUC	Brier	ECE	HarmRS
PRR	LR	CDDD	53 ± 9	53 ± 9	54 ± 3	62 ± 4	29 ± 4	17 ± 7	51 ± 3
		ChemBERTa	50 ± 7	52 ± 6	51 ± 5	59 ± 5	30 ± 4	20 ± 6	50 ± 3
		Klek	52 ± 9	58 ± 8	57 ± 4	64 ± 4	27 ± 3	13 ± 6	54 ± 4
		MACCS	50 ± 8	57 ± 8	55 ± 2	63 ± 3	27 ± 2	13 ± 4	52 ± 3
		ECFP	51 ± 10	58 ± 7	56 ± 5	63 ± 4	28 ± 3	15 ± 7	53 ± 5
		RDKit	53 ± 7	55 ± 7	56 ± 4	64 ± 3	27 ± 2	13 ± 5	54 ± 3
	RF	CDDD	60 ± 11	51 ± 11	58 ± 4	66 ± 5	24 ± 1	7 ± 3	53 ± 5
		ChemBERTa	59 ± 11	46 ± 11	54 ± 4	62 ± 3	25 ± 1	7 ± 3	50 ± 5
		Klek	46 ± 11	64 ± 11	57 ± 3	65 ± 3	25 ± 1	10 ± 4	52 ± 5
		MACCS	56 ± 11	54 ± 8	58 ± 3	66 ± 3	25 ± 1	8 ± 3	54 ± 4
		ECFP	48 ± 11	63 ± 10	58 ± 3	66 ± 3	25 ± 1	10 ± 3	53 ± 5
		RDKit	60 ± 9	51 ± 8	58 ± 4	65 ± 4	25 ± 1	7 ± 3	54 ± 3
	XGB	CDDD	58 ± 8	54 ± 7	58 ± 3	65 ± 3	25 ± 1	10 ± 3	55 ± 2
		ChemBERTa	56 ± 7	52 ± 7	55 ± 3	63 ± 3	26 ± 1	12 ± 2	53 ± 2
		Klek	53 ± 9	59 ± 8	58 ± 3	65 ± 3	26 ± 1	11 ± 3	54 ± 4
		MACCS	53 ± 7	59 ± 7	58 ± 3	66 ± 3	25 ± 1	11 ± 3	55 ± 3
		ECFP	54 ± 10	58 ± 7	58 ± 4	65 ± 3	26 ± 2	11 ± 4	55 ± 4
		RDKit	56 ± 8	56 ± 7	59 ± 4	66 ± 4	25 ± 1	10 ± 3	55 ± 2
ROR	LR	CDDD	50 ± 8	57 ± 7	54 ± 4	62 ± 3	30 ± 3	21 ± 5	52 ± 3
		ChemBERTa	49 ± 7	54 ± 6	53 ± 4	60 ± 5	31 ± 2	22 ± 4	51 ± 3
		Klek	49 ± 6	59 ± 7	56 ± 4	63 ± 4	29 ± 2	18 ± 4	53 ± 3
		MACCS	39 ± 6	67 ± 7	54 ± 3	62 ± 3	28 ± 1	17 ± 2	48 ± 4
		ECFP	50 ± 8	57 ± 7	55 ± 5	62 ± 4	29 ± 2	19 ± 4	52 ± 4
		RDKit	41 ± 8	67 ± 7	56 ± 4	64 ± 4	28 ± 2	18 ± 4	50 ± 5
	RF	CDDD	56 ± 9	55 ± 8	58 ± 4	66 ± 4	24 ± 1	8 ± 3	55 ± 3
		ChemBERTa	54 ± 8	53 ± 8	55 ± 3	63 ± 3	25 ± 1	8 ± 3	52 ± 3
		Klek	37 ± 8	72 ± 7	57 ± 3	65 ± 4	26 ± 1	13 ± 3	48 ± 6
		MACCS	55 ± 9	57 ± 6	59 ± 4	67 ± 4	25 ± 1	8 ± 3	55 ± 3
		ECFP	36 ± 8	74 ± 7	57 ± 3	65 ± 3	26 ± 1	13 ± 4	47 ± 6
		RDKit	55 ± 9	56 ± 6	58 ± 4	65 ± 4	25 ± 1	8 ± 3	55 ± 3
	XGB	CDDD	52 ± 8	59 ± 8	58 ± 3	66 ± 3	25 ± 1	10 ± 3	54 ± 3
		ChemBERTa	52 ± 7	56 ± 6	56 ± 4	64 ± 3	26 ± 1	11 ± 3	53 ± 3
		Klek	47 ± 8	64 ± 7	57 ± 3	65 ± 3	27 ± 1	13 ± 3	53 ± 4
		MACCS	48 ± 7	64 ± 6	58 ± 3	66 ± 3	26 ± 1	12 ± 3	54 ± 4
		ECFP	49 ± 9	63 ± 6	58 ± 5	65 ± 4	26 ± 2	13 ± 4	54 ± 5
		RDKit	52 ± 7	61 ± 6	59 ± 4	66 ± 4	26 ± 1	11 ± 3	55 ± 3
IC	LR	CDDD	61 ± 8	44 ± 7	53 ± 4	61 ± 3	31 ± 3	22 ± 4	50 ± 3
		ChemBERTa	60 ± 7	42 ± 6	52 ± 5	59 ± 5	31 ± 3	22 ± 5	49 ± 3
		Klek	63 ± 7	42 ± 6	54 ± 4	62 ± 4	30 ± 3	21 ± 4	50 ± 3
		MACCS	75 ± 6	28 ± 5	54 ± 3	62 ± 3	28 ± 1	16 ± 2	40 ± 5
		ECFP	62 ± 7	44 ± 5	54 ± 6	61 ± 5	30 ± 3	21 ± 6	51 ± 4
		RDKit	71 ± 7	34 ± 7	56 ± 4	64 ± 4	28 ± 2	17 ± 3	45 ± 6
	RF	CDDD	78 ± 11	31 ± 14	59 ± 3	66 ± 4	24 ± 1	5 ± 2	41 ± 13
		ChemBERTa	77 ± 10	29 ± 13	57 ± 2	64 ± 3	24 ± 1	5 ± 2	40 ± 12
		Klek	76 ± 12	31 ± 13	58 ± 3	65 ± 4	25 ± 1	8 ± 3	41 ± 10
		MACCS	73 ± 10	37 ± 12	59 ± 3	66 ± 3	24 ± 1	6 ± 2	47 ± 8
		ECFP	74 ± 11	33 ± 12	58 ± 4	66 ± 4	24 ± 1	7 ± 3	43 ± 9
		RDKit	75 ± 10	34 ± 11	59 ± 4	66 ± 4	24 ± 1	5 ± 2	45 ± 9
	XGB	CDDD	74 ± 8	34 ± 6	57 ± 3	65 ± 3	26 ± 1	13 ± 2	45 ± 5
		ChemBERTa	74 ± 6	31 ± 7	55 ± 3	63 ± 3	27 ± 1	14 ± 3	43 ± 6
		Klek	74 ± 7	35 ± 7	58 ± 3	65 ± 4	26 ± 1	13 ± 2	46 ± 5
		MACCS	74 ± 6	34 ± 6	58 ± 2	66 ± 3	26 ± 1	12 ± 2	46 ± 5
		ECFP	72 ± 9	36 ± 7	58 ± 5	65 ± 4	26 ± 2	13 ± 3	47 ± 4
		RDKit	74 ± 7	34 ± 7	58 ± 3	66 ± 4	26 ± 1	13 ± 3	46 ± 5

2.6. Class and label weights distributions

Table S6. Class and label weights distributions. Mean LW - Mean Label Weight, Adj. Fraction - Fraction of compounds adjusted by Label Weights.

Category	Sub-category	Class	Entries	Mean LW	Fraction	Adj. Fraction
Sex	Male	Toxic	4312	0.266	54.60	43.10
		Non-Toxic	3579	0.423	45.40	56.90
	Female	Toxic	4884	0.263	59.60	48.60
		Non-Toxic	3317	0.410	40.40	51.40
	Unknown	Toxic	5985	0.294	58.80	49.00
		Non-Toxic	4196	0.436	41.20	51.00
Age	Children	Toxic	1297	0.166	67.38	51.30
		Non-Toxic	628	0.325	32.62	48.70
	Adolescent	Toxic	1316	0.181	69.74	55.47
		Non-Toxic	571	0.334	30.26	44.53
	Adult	Toxic	4073	0.271	56.70	45.84
		Non-Toxic	3110	0.420	43.30	54.16
	Elderly	Toxic	3324	0.287	52.08	42.28
		Non-Toxic	3059	0.426	47.92	57.72
	Unknown	Toxic	5171	0.324	58.13	49.67
		Non-Toxic	3724	0.456	41.87	50.33
Weight	Low	Toxic	3073	0.213	56.84	41.99
		Non-Toxic	2333	0.387	43.16	58.01
	Average	Toxic	3188	0.220	57.98	43.35
		Non-Toxic	2310	0.397	42.02	56.65
	High	Toxic	2813	0.215	55.45	40.23
		Non-Toxic	2260	0.398	44.55	59.77
	Unknown	Toxic	6107	0.365	59.31	52.91
		Non-Toxic	4189	0.473	40.69	47.09

2.7. Correlation between absolute error and minimum Tanimoto distance to training folds

Table S7. Correlation between absolute error and minimum Tanimoto distance to training folds.

Model	Pearson r	P_{value}
No-demographic	0.017	0.56
Sex-only	0.094	< 0.001
Age-only	0.079	< 0.001
Weight-only	0.091	< 0.001
Combined-demographic	0.093	< 0.001

2.8. Missing and removed entries by demographic subpopulation

Table S8. Percentages of missing and removed entries by demographic subpopulation.

Demographics	%Missing	%Removed
Sex	12.80	0.00
Age	42.83	0.19
Weight	81.20	11.75

2.9. Overview of used molecular descriptors

Table S9. Molecular descriptors used in the study.

Name	Type	Size
MACCS [1]	Fragment	166
Klekota & Roth [2]	Fragment	4860
RDKit [3]	Topological	210
ECFP [4]	Circular	1024
CDDD [5]	Embedding	512
ChemBERTa [6]	Embedding	384

MACCS and Klekota&Roth fingerprints are fragment-based binary vectors, where each bit indicates the presence (1) or absence (0) of predefined molecular substructures. MACCS fingerprints were designed primarily for drug discovery and development, while Klekota&Roth fingerprints were tailored for molecular similarity and activity comparison tasks. RDKit descriptors belong to the class of topological descriptors that quantify various molecular properties derived directly from molecular graphs. Extended-Connectivity Fingerprints (ECFP), a subclass of circular fingerprints, encode numerically represented sets of molecular substructures using a hashing function to generate fixed-length binary vectors. CDDD descriptors are 512-dimensional continuous vectors obtained from an encoder-decoder neural network trained to translate SMILES strings into InChI representations. ChemBERTa embeddings are 384-dimensional vectors generated by a transformer-based language model pre-trained on a large corpus of SMILES strings.

2.10. Hyperparameter optimization space

Table S10. Optimized hyperparameters and their permitted values.

Parameter	Range/Options	Type	Scale
	Random Forest		
n_estimators	100 - 1000 (step: 25)	Integer	Linear
max_depth	2 - 20	Integer	Linear
min_samples_leaf	1 - 10	Integer	Linear
min_samples_split	2 - 10	Integer	Linear
max_features	sqrt, log2, None	Categorical	-
criterion	gini, entropy, log_loss	Categorical	-
ccp_alpha	1e-5 - 0.05	Float	Logarithmic
	XGBoost		
n_estimators	100 - 1000 (step: 25)	Integer	Linear
max_depth	2 - 12	Integer	Linear
max_leaves	0 - 12	Integer	Linear
learning_rate	5e-3 - 1e-1	Float	Logarithmic
subsample	0.5 - 1.0	Float	Linear
colsample_bytree	0.5 - 1.0	Float	Linear
gamma	0 - 5	Float	Linear
reg_alpha	0 - 5	Float	Linear
reg_lambda	0 - 5	Float	Linear
	Logistic Regression		
solver*	lbfgs, liblinear, newton-cg, newton-cholesky, sag, saga	Categorical	-
penalty*	l1, l2, elasticnet, None	Categorical	-
C	0.001 - 10	Float	Logarithmic
max_iter	1024	Integer	Fixed
l1_ratio**	0.0 - 1.0	Float	Linear

* Solvers and Penalties were sampled together from a set of permitted combinations.
** Only used when penalty was set to elasticnet.

3. Methodological Details

3.1. FAERS processing and mapping algorithm

Relevant drug descriptions were obtained by filtering MedDRA (version 27.1)[7] Preferred Terms (PTs) using selected High-Level Terms (HLT) from the System Organ Class (SOC) categories: "Cardiac disorders", "Investigations", "General disorders and administration site conditions", and "Vascular disorders". All initially qualified terms were then manually checked. The irrelevant ones, including congenital, infectious and injury-related conditions, were removed.

The prepared database was initially queried to retrieve entries where at least one reported reaction was relevant. The resulting subset contained around 1.2 million patients and around 150,000 unique drug descriptions. Non-English characters that were not correctly encoded were replaced with question marks, characters used for separation of individual drugs (e.g., comma, hyphen, backslash) were standardized and represented using ":", non-alphanumeric characters were replaced with white spaces, and details on dosage, administration frequency, route, and pharmaceutical form were removed. The processed entries were divided into individual drug descriptions and assigned to active ingredients using Algorithm S1, presented below.

Algorithm S1 Mapping from drug descriptions to active ingredients.

```
procedure PROCESS TOKENS(tokens)
    for token in tokens do
        if token in DrugBank then
            capture
        else
            calculate similarity to drugs and synonyms in DrugBank
            calculate similarity to previous tokens
            query external databases                    ▷ e.g. PubChem, PME, RxReasoner

            decision ← input()
            if decision == "remove" then
                remove token
            else if decision == "substitute" then
                replace token with user-provided string
            else if decision == "update" then
                add new information to the token
            else if decision == "capture" then
                capture
            else
                skip
            end if
        end if
    end for
end procedure
```

Eighty-five percent of the processed entries were assigned to the corresponding active ingredients. Only entries containing valid drugs or clear combinations were retained. The following groups were removed: vaccines, immunoglobulins, RNA-based drugs, peptides, proteins, polymers, probiotics, herbal and homeopathic formulations, infusion or dialysis fluids, multivitamins, foods, nutritional preparations, abbreviations (unless they represented a clear combination, e.g., "smz-tmp" as sulfamethoxazole and trimethoprim), and entries for which contradictory results were returned (unless a majority of sources favored one option), among others.

To extend the mapping, we performed full record linkage between the initially selected drug de-

scriptions and all remaining ones. Matching was performed on the basis of string similarity using the Damerau-Levenshtein distance. Following manual inspection, pairs with a similarity score below 0.85 were removed. The final mapping of drug description-to-active ingredient comprised 311,451 drug descriptions, 8260 drug combinations, and 4333 unique drugs.

3.2. Disproportionality Analysis

	Reaction	No Reaction
Drug	a	b
No Drug	c	d

Figure S8. Confusion matrix for DPA assignments.

The PRR is calculated as:

$$PRR = \frac{a \,/\, (a+b)}{c \,/\, (c+d)} \tag{1}$$

The lower bound of the Confidence Interval is calculated using the delta method derived by C. Gravel[8]:

$$PRR_{\alpha/2} = PRR \cdot \exp\big(Q_Z(\alpha/2) \cdot s\big), \text{ where:} \tag{2}$$

$Q_Z(\alpha/2)$ is the quantile function of the standard normal distribution evaluated at $\alpha/2$, and s is the estimated stan

$$s = \sqrt{\frac{1}{a} - \frac{1}{a+b} + \frac{1}{c} - \frac{1}{c+d}} \tag{3}$$

The ROR is calculated as:

$$ROR = \frac{a \,/\, b}{c \,/\, d} \tag{4}$$

The lower bound of the Confidence Interval is calculated similarly to the PRR:

$$ROR_{\alpha/2} = ROR \cdot \exp\big(Q_Z(\alpha/2) \cdot s\big), \text{ where:} \tag{5}$$

$$s = \sqrt{\frac{1}{a} + \frac{1}{b} + \frac{1}{c} + \frac{1}{d}} \tag{6}$$

The IC is calculated as:

$$IC = \log_2\big(\frac{a+\kappa}{N_{exp}+\kappa}\big), \text{ where:} \tag{7}$$

κ is a shrinkage factor set to 0.5, N_{exp} is the expected number of records calculated as:

$$N_{exp} = \frac{(a+b) \cdot (a+c)}{a+b+c+d} \tag{8}$$

The lower bound of the Confidence Interval is calculated using a conjugate gamma distribution:

$$\Gamma = \Gamma(shape = a+\kappa, rate = \frac{1}{N'_{exp}+\kappa}) \tag{9}$$

$$IC_{\alpha/2} = \log_2\big(Q_\Gamma(\alpha/2)\big), \text{ where:} \tag{10}$$

$Q_\Gamma(\alpha/2)$ is the quantile function of the gamma distribution evaluated at $\alpha/2$. The upper bounds of Confidence Intervals for all metrics were calculated in a similar way, but using the corresponding quantile functions evaluated at $1-\alpha/2$. Throughout the study, we used the α value of 0.01.

3.3. Label assignment

For each compound, we assigned a confidence score based on the distance of the calculated metric (μ) to the threshold (T) and the range of the confidence interval.

$$\text{Confidence score} = \frac{\sqrt{|\ \mu - T\ |}}{CI_{upper} - CI_{lower}} \tag{11}$$

The calculated Confidence scores are defined in the [0, $+\infty$] range. We used a modified sigmoid function—given in Equation 12 and shown in Figure S9—that maps the inputs to the [0.1, 1.0] interval.

$$y = f(x) = \frac{1}{1 + e^{-\frac{4}{5}\log 9 \cdot (x - \frac{5}{4})}} \tag{12}$$

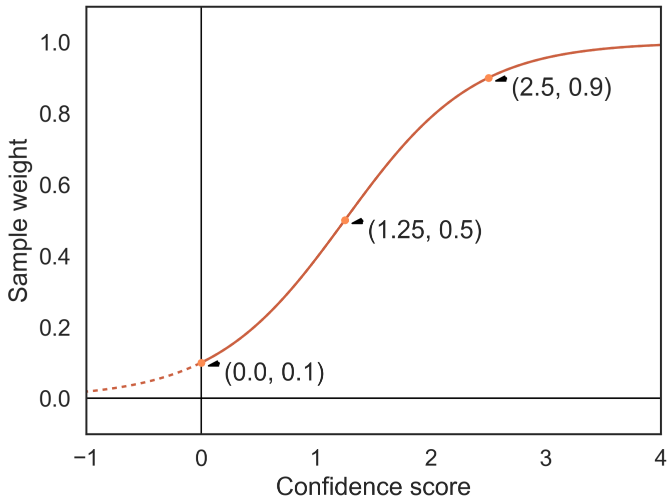

Figure S9. The shape and selected points of the modified sigmoid function.

3.4. Evaluation Metrics

The following metrics were used throughout the study to measure the performance of trained machine learning models. TP, TN, FP, and FN denote the number of true positive, true negative, false positive, and false negative classifications, respectively.

- **Accuracy** measures the proportion of correctly predicted entries:

$$\text{Accuracy} = \frac{TP + TN}{TP + TN + FP + FN} \tag{13}$$

- **Recall** (True Positive Rate (TPR), Sensitivity) measures the proportion of positive instances that were predicted to be positive:

$$\text{Recall} = \frac{TP}{TP + FN} \tag{14}$$

- **Specificity** (True Negative Rate (TNR)) measures the proportion of negative instances that were predicted to be negative:

$$\text{Specificity} = \frac{TN}{TN + FP} \tag{15}$$

- **Precision** measures the proportion of positive instances among all entries predicted to be positive:

$$\text{Precision} = \frac{TP}{TP + FP} \tag{16}$$

- **False Positive Rate (FPR)** measures the proportion of negative instances that were incorrectly predicted to be positive:

$$\mathrm{FPR} = \frac{FP}{FP + TN} \tag{17}$$

- **Harmonic mean of Recall and Specificity (HarmRS)** provides a balanced summary of recall and specificity and penalizes models that perform well on only one metric:

$$\mathrm{HarmRS} = \frac{2 \cdot \mathrm{Recall} \cdot \mathrm{Specificity}}{\mathrm{Recall} + \mathrm{Specificity}} \tag{18}$$

- **ROC AUC** is the Area Under the Receiver Operating Characteristic Curve, which plots recall against the False Positive Rate across all classification thresholds[9]. More intuitively, it is equivalent to the probability that a randomly chosen positive entry is ranked higher than a randomly chosen negative:

$$\mathrm{ROC\ AUC} = \int_0^1 \mathrm{TPR}(t)\, d(\mathrm{FPR}(t)) \tag{19}$$

- **PRC AUC** is the Area Under the Precision-Recall Curve, which plots precision against recall across all classification thresholds and provides a balanced estimate of model performance under class imbalance[10]:

$$\mathrm{PRC\ AUC} = \int_0^1 \mathrm{Precision}(r)\, d(r) \tag{20}$$

- **Brier score** measures the mean squared error between predicted probabilities ($\hat{p}_i$) and true binary outcomes (y_i), with lower values corresponding to better probabilistic predictions[11]:

$$\mathrm{BS} = \frac{1}{N} \sum_{i=1}^{N} (\hat{p}_i - y_i)^2 \tag{21}$$

- **Expected Calibration Error (ECE)** measures the average discrepancy between predicted confidence and observed accuracy across M evenly spaced probability bins B_m[12]:

$$\mathrm{ECE} = \sum_{m=1}^{M} \frac{|B_m|}{N} \cdot |\mathrm{Acc}(B_m) - \mathrm{Conf}(B_m)|, \tag{22}$$

  where $\mathrm{Acc}(B_m)$ and $\mathrm{Conf}(B_m)$ denote the mean accuracy and mean predicted probability within bin B_m, respectively.

4. References

[1] J. L. Durant, B. A. Leland, D. R. Henry, and J. G. Nourse, “Reoptimization of MDL keys for use in drug discovery,” *J. Chem. Inf. Comput. Sci.*, vol. 42, pp. 1273–1280, Nov. 2002.

[2] J. Klekota and F. P. Roth, “Chemical substructures that enrich for biological activity,” *Bioinformatics*, vol. 24, no. 21, pp. 2518–2525, 2008.

[3] G. Landrum, P. Tosco, B. Kelley, R. Rodriguez, D. Cosgrove, R. Vianello, sriniker, gedeck, G. Jones, NadineSchneider, E. Kawashima, D. Nealschneider, A. Dalke, M. Swain, B. Cole, S. Turk, A. Savelev, A. Vaucher, M. Wójcikowski, I. Take, V. F. Scalfani, R. Walker, K. Ujihara, D. Probst, guillaume godin, A. Pahl, J. Lehtivarjo, F. Berenger, jasondbiggs, and strets123, “rdkit/rdkit: 2024-03-1 (q1 2024) release,” 2024.

[4] H. L. Morgan, “The generation of a unique machine description for chemical structures-a technique developed at chemical abstracts service,” *J. Chem. Doc.*, vol. 5, pp. 107–113, May 1965.

[5] R. Winter, F. Montanari, F. Noé, and D.-A. Clevert, “Learning continuous and data-driven molecular descriptors by translating equivalent chemical representations,” *Chemical Science*, vol. 10, pp. 1692–1701, 2019.

[6] S. Chithrananda, G. Grand, and B. Ramsundar, “ChemBERTa: Large-scale self-supervised pretraining for molecular property prediction,” *arXiv*, Oct. 2020.

[7] E. G. Brown, L. Wood, and S. Wood, “The medical dictionary for regulatory activities (MedDRA),” *Drug Saf.*, vol. 20, pp. 109–117, Feb. 1999.

[8] C. Gravel, *Statistical methods for signal detection in pharmacovigilance.* PhD thesis, Carleton University, 2009.

[9] J. A. Hanley and B. J. McNeil, “The meaning and use of the area under a receiver operating characteristic (roc) curve.,” *Radiology*, vol. 143, p. 2936, Apr. 1982.

[10] J. Davis and G. Mark, “The relationship between precision-recall and roc curves,” in *Proceedings of the 23rd International Conference on Machine Learning*, 2006.

[11] G. W. BRIER, “Verification of forecasts expressed in terms of probability,” *Monthly Weather Review*, vol. 78, p. 13, Jan. 1950.

[12] C. Guo, G. Pleiss, and K. Q. Weinberger, “On calibration of modern neural networks,” in *Proceedings of the 34th International Conference on Machine Learning*, 2017.
